# Red Blood Cell Membrane‐Coated Nanoparticles Enable Incompatible Blood Transfusions

**DOI:** 10.1002/advs.202310230

**Published:** 2024-06-05

**Authors:** Xuewei Yang, Mengchun Chen, Cuiye Weng, Deli Zhuge, Fangsi Jin, Yingnan Xiao, Dongyan Tian, Qingqing Yin, Li Li, Xufei Zhang, Genghe Shi, Xiaosheng Lu, Linzhi Yan, Ledan Wang, Bin Wen, Yingzheng Zhao, Jiajin Lin, Fang Wang, Weixi Zhang, Yijie Chen

**Affiliations:** ^1^ Department of Obstetrics and Gynecology The Second Affiliated Hospital of Wenzhou Medical University Wenzhou 325027 China; ^2^ Department of Pharmacy The Second Affiliated Hospital of Wenzhou Medical University Wenzhou 325027 China; ^3^ Department of Pharmaceutics School of Pharmaceutical Sciences of Wenzhou Medical University Wenzhou 325035 China; ^4^ Department of Pediatric Allergy and Immunology The Second Affiliated Hospital of Wenzhou Medical University Wenzhou 325027 China; ^5^ Department of Blood Transfusion The Second Affiliated Hospital of Wenzhou Medical University Wenzhou 325027 China; ^6^ Wenzhou Medical University Wenzhou 325027 China; ^7^ Cixi Biomedical Research Institute Wenzhou Medical University Ningbo 315302 China

**Keywords:** antibody, biomimetic nanoparticle, cell membrane, incompatible blood transfusion, neutralization

## Abstract

Blood transfusions save lives and improve health every day. Despite the matching of blood types being stricter than it ever has been, emergency transfusions among incompatible blood types are still inevitable in the clinic when there is a lack of acceptable blood types for recipients. Here to overcome this, a counter measure nanoplatform consisting of a polymeric core coated by a red blood cell (RBC) membrane is developed. With A‐type or B‐type RBC membrane camouflaging, the nanoplatform is capable of specifically capturing anti‐A or anti‐B IgM antibodies within B‐type or A‐type whole blood, thereby decreasing the corresponding IgM antibody levels and then allowing the incompatible blood transfusions. In addition to IgM, the anti‐RBC IgG antibody in a passive immunization murine model can likewise be neutralized by this nanoplatform, leading to prolonged circulation time of incompatible donor RBCs. Noteworthily, nanoplatform made by expired RBCs (>42 days stored hypothermically) and then subjected to lyophilization does not impair their effect on antibody neutralization. Most importantly, antibody‐captured RBC‐NP do not exacerbate the risk of inflammation, complement activation, and coagulopathy in an acute hemorrhagic shock murine model. Overall, this biomimetic nanoplatform can safely neutralize the antibody to enable incompatible blood transfusion.

## Graphical abstract

Red blood cell RBC(A)‐nanoparticle (NP) made by RBC(A)s could neutralize anti‐A immunoglobulin M (IgM) antibodies in wRBC(B), leading to decreased anti‐A IgM levels, and this then allowed the transfusion of incompatible RBC(A)s or RBC(AB)s. This strategy also could be leveraged to make acceptable blood transfusions between wRBC(A) and RBC(B)s or RBC(AB)s or between wRBC(O) and RBC(A)s, RBC(B)s, or RBC(AB)s. Additionally, the anti‐RBC IgG antibody could likewise be neutralized by this nanoplatform.

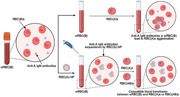

## 1 Introduction

Transfusion of blood, or more commonly red blood cells (RBCs), saves lives if patients have lost blood from an injury or during surgery or are facing life‐threatening medical conditions.^[^ ^1^ ^]^ However, the imbalanced blood supply, insufficient blood bank inventory, short validity period, and improper preservation of blood bags are seriously threatening the supply of blood.^[^ ^2^ ^]^ Given these reasons, the blood supply cannot always meet demand, particularly in hospitals' emergency centers that are often asked for a larger need.^[^ ^3^ ^]^ In the clinic, methods such as developing a national blood management policy or extending the storage time of RBCs are usually employed to maintain a high reservation status of blood banks.^[^ ^4^, ^5^ ^]^ Additionally, blood type matching must be considered during blood transfusions, further aggravating the shortage of blood supply.^[^ ^6^ ^]^ Therefore, overcoming incompatible blood transfusions is vital for saving lives and lessening the tight blood supply worldwide.

Most strategies for addressing incompatible blood transfusion center around converting or shielding incompatible antigens on the surface of RBCs. One such method has been enzyme treatment, which aims to convert the A, B, or AB‐type RBCs (denote RBC(A)s, RBC(B)s, or RBC(AB)s) to O‐type RBCs (denote RBC(O)s) by employing specific enzymes.^[^ ^7^, ^8^ ^]^ Despite effectiveness, enzymes make the process expensive, and the incompatibility issues upon converted RBCs remain. Another method is to confer RBCs a molecular “stealth” that helps to block the incompatible antigens on the surface of RBCs from the immune system recognition.^[^ ^9^, ^10^, ^11^ ^]^ Unfortunately, this process not only potentially generates chemical contamination and decreases the cell membrane fluidity but also may compromise the RBC's role as an immunomodulator.^[^ ^12^ ^]^ Besides these, the maximum shelf‐life of RBCs, regardless of A, B, AB, or O blood types, is 42 d^[^ ^13^ ^]^; such a short storage window impedes strategic options around modifying incompatible RBCs for blood transfusions.

**Scheme 1:**
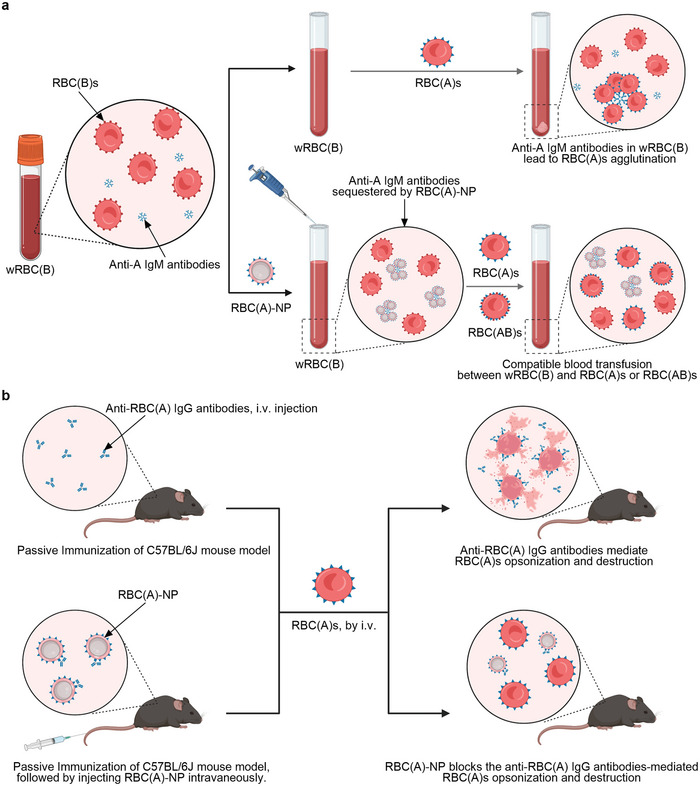
a) RBC(A)‐NP made by RBC(A)s could be utilized to neutralize anti‐A IgM antibodies in wRBC(B), leading to decreased anti‐A IgM levels, and this then allowed the transfusion of incompatible RBC(A)s or RBC(AB)s. This strategy also could be leveraged to make acceptable blood transfusions between wRBC(A) and RBC(B)s or RBC(AB)s or between wRBC(O) and RBC(A)s, RBC(B)s, or RBC(AB)s. b) Similarly, RBC(A)‐NP could be employed to block the destructive anti‐RBC(A) IgG‐induced RBC(A)s opsonization and clearance in a passive immunization murine model.

More recently, cell membrane‐camouflaged nanoparticles have emerged for wide biomedical applications due to their biomimicry of source cells.^[^ ^14^, ^15^, ^16^, ^17^, ^18^, ^19^, ^20^, ^21^ ^]^ By a top‐down approach, it has previously been shown that the natural cytoplasmic membrane of RBCs, particularly the transmembrane protein repertoire including antigens involved in incompatible blood transfusion, could be faithfully translocated onto nanoparticles.^[^ ^22^ ^]^ Inspired by such unique biomimicry, herein, we developed an RBC membrane‐coated PLGA (poly(lactic‐co‐glycolic) acid) nanoparticle (RBC‐NP) capable of mimicking real RBCs for counteracting the corresponding antibodies, including IgM and IgG. In this study, RBC(A)‐NP made by RBC(A)s could be leveraged to capture anti‐A IgM antibodies in B‐type whole blood (denoted wRBC(B)), leading to decreased free anti‐A IgM amounts and this then allowed the transfusion of incompatible RBC(A)s or RBC(AB)s (**Scheme** **1**a). Similarly, the broad applicability of this strategy held true when we utilized RBC(B)‐NP (made by RBC(B)s) to eliminate anti‐B IgM antibodies in A‐type whole blood (denoted wRBC(A)s) or RBC(A)‐NP combined with RBC(B)‐NP to simultaneously sequester anti‐A and anti‐B IgM antibodies in O‐type whole blood (denoted wRBC(O)s). The resultant decreased IgM antibody levels led to successful blood transfusions between wRBC(A)s and RBC(B)s or RBC(AB)s, or between wRBC(O)s and RBC(A)s, RBC(B)s, or RBC(AB)s. In addition to IgM, RBC(A)‐NP likewise was able to neutralize the destructive anti‐RBC(A) IgG antibodies to preserve incompatible RBC(A)s for prolonged circulation time in a passive immunization murine model (Scheme 1b). Noteworthily, the RBC‐NP formulated with the membrane from expired RBCs exhibited a comparable neutralization effect against IgM/IgG antibodies to those nanoparticles made by fresh RBCs, and after lyophilization and resuspension also did not decrease their efficacy. Furthermore, we confirmed that IgM or IgG antibodies‐sequestered RBC‐NP were preferentially biodistributed at the liver and spleen, and they did not negatively impact the state of the body in an acute hemorrhagic shock murine model. Overall, these results demonstrated that RBC‐NP conferred a generalized and safe strategy for overcoming incompatible blood transfusion by neutralizing antibodies regarding incompatible blood transfusions. This method was promising for clinical applications when we cannot afford enough compatible RBCs for blood transfusion in emergencies.

## 2 Results and Discussion

### 2.1 Preparation and Characterization of RBC‐NP That Made by RBC(A)s and RBC(B)s

**Figure 1:**
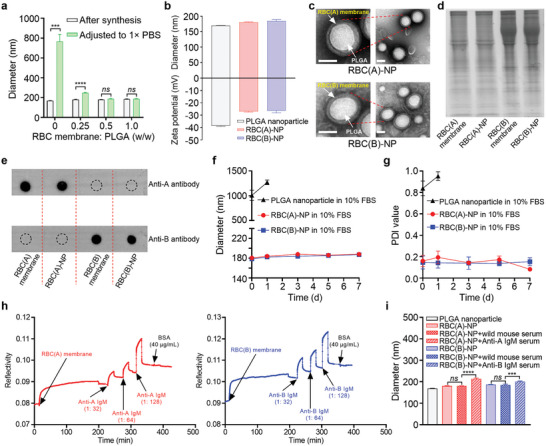
Preparation and characterization of RBC‐NP. a) Hydrodynamic sizes of RBC‐NP formulated with various RBC membranes to PLGA nanoparticles after synthesis in water and after adjusting to 1× PBS. (*n* = 3, mean ± SD). b) Diameters and zeta potentials of PLGA nanoparticle, RBC(A)‐NP, and RBC(B)‐NP after synthesis. RBC(A)‐NP and RBC(B)‐NP were synthesized at a weight ratio of RBC membrane to PLGA nanoparticle of 0.5: 1. (*n* = 3, mean ± SD). c) TEM images of RBC(A)‐NP and RBC(B)‐NP (scale bar = 100 nm). d) SDS‐PAGE protein analysis of RBC(A) membrane, RBC(A)‐NP, RBC(B) membrane, and RBC(B)‐NP at an equivalent protein concentration. All samples were imaged after Coomassie Blue staining. e) Antigen A expressed in RBC(A) membrane and RBC(A)‐NP or antigen B expressed in RBC(B) membrane and RBC(B)‐NP as determined by Dot blot. f) The colloidal stability and g) monodispersity of PLGA nanoparticle, RBC(A)‐NP, and RBC(B)‐NP in 10% FBS were monitored over 7 d. (*n* = 3, mean ± SD). h) The interactions between RBC(A) membrane and varying titers of anti‐A IgM serums (1: 32, 1: 64, and 1: 128 titers) or between RBC(B) membrane and varying titers of anti‐B IgM serums (1: 32, 1: 64, and 1: 128 titers), as measured by SPR. i) Diameter changes of RBC(A)‐NP or RBC(B)‐NP before and after the addition of the corresponding anti‐A (1: 128 titers) or anti‐B (1: 128 titers) IgM serums. RBC(A)‐NP or RBC(B)‐NP treated with wild mouse serums were used as controls. (*n* = 3, mean ± SD).

Prior to fabricating the RBC‐NP, we first derived RBC(A) and RBC(B) membranes from packed RBC(A)s and RBC(B)s through a hypotonic treatment following previous works. Meanwhile, polymeric PLGA nanoparticles were synthesized via a nanoprecipitation method. Then, RBC(A) and RBC(B) membranes were coated onto PLGA cores to form respective RBC(A)‐NP and RBC(B)‐NP by a sonication process.^[^ ^23^, ^24^, ^25^ ^]^ To determine an optimal ratio, we prepared a series of samples containing various amounts of RBC membrane (as expressed in terms of RBC membrane protein) but a fixed amount of PLGA nanoparticle followed by sonication. At ratios of 0.5 mg membrane per 1 mg PLGA nanoparticle or beyond, the hydrodynamic diameters of RBC‐NP remained consistent when particles were transferred from water to PBS. In contrast, at a lower ratio of 0.25, the incomplete membrane coverage upon PLGA nanoparticle could be readily impacted by charge screening in ionic buffer, which resulted in significantly increased size and polydispersity index (PDI) values in PBS (**Figures** **1**a and S1, Supporting Information). Thus, we selected the membrane/PLGA ratio of 0.5 (mg mg^−1^) to fabricate RBC‐NP thereafter. Upon this ratio, the resulting RBC(A)‐NP and RBC(B)‐NP with a comparable size of about 180 nm exhibited 20 nm larger than bare PLGA nanoparticle, corresponding to confer a bilayered cell membrane thickness onto polymeric cores. Additionally, these biomimetic nanoparticles had an equivalent zeta potential of approximately ‐26 mV that were all higher compared to that of PLGA nanoparticle alone, again suggesting the successful membrane coating (Figure 1b). To visualize the membrane coating, transmission electron microscopy (TEM) imaging of RBC(A)‐NP and RBC(B)‐NP revealed PLGA cores enclosed inside the RBC membrane (Figure 1c) compared to bare PLGA (Figure S2, Supporting Information); meanwhile, fluorescence images showing 1,1′‐dioctadecyl‐3,3,3′,3′‐tetramethylindodicarbocyanine 4‐chlorobenzenesulfonate salt (DiD) dye labeled RBC membrane in red color overlapped with 3,3‐Dioctadecyloxacarbocyanine perchlorate (DiO) dye labeled PLGA core at nearly 100% efficiency were photographed by confocal microscopy (Figure S3, Supporting Information). Such successful coating could faithfully copy complete cell membrane protein compositions from RBC(A) and RBC(B) membranes to RBC(A)‐NP and RBC(B)‐NP respectively, as detected by sodium dodecyl sulfate‐polyacrylamide gel electrophoresis (SDS‐PAGE) (Figure 1d). Furthermore, the incompatible blood transfusion‐related antigens on RBC(A)‐NP and RBC(B)‐NP, including A and B antigens respectively, were characterized by Dot blot (Figure 1e) and Western blot Figure S4, Supporting Information). Also, the RBC membrane cloaking was effective at providing PLGA nanoparticle with excellent colloidal stability and monodispersity in 10% FBS over 7 d, as corroborated by unchanged morphology at day 7 (Figure S5, Supporting Information); otherwise, PLGA nanoparticle would aggregate quickly to microscale (Figure 1f,g).

After having confirmed the PLGA cores coated with the RBC membrane, we next aimed to investigate whether the obtained RBC‐NP could interact with the corresponding IgM antibodies. By employing a surface plasmon resonance (SPR) method, the RBC(A) membrane could respond to anti‐A IgM serum at an increasing range from 1:32 to 1:128 titers, and the same result held true when RBC(B) membrane was leveraged to test its affinity to anti‐B IgM serum (Figure 1h). In addition, by interacting with serums carrying the corresponding IgM antibodies, both RBC(A)‐NP and RBC(B)‐NP had a significant increase in diameters compared to that of RBC‐NP only, or that of RBC‐NP treated by wild mouse serum (Figure 1i). These results strongly suggested that RBC(A)‐NP and RBC(B)‐NP, with inherited properties of source RBCs, were likely capable of capturing the corresponding IgM antibodies.

### 2.2 RBC‐NP Blocked the Corresponding IgM‐Mediated Incompatible Blood Transfusions

Once incompatible blood is given in a transfusion, the recipient's immune system detects and tags donor RBCs as invaders and starts to clear them up. This process not only makes incompatible blood useless, but the recipient's body would also be overwhelmed by the overactivated immune response.^[^ ^26^ ^]^ When looking at such incompatible blood transfusion reactions, they are often initiated by the antibodies of recipients that bind at donor RBCs and are followed by complement activation.^[^ ^27^ ^]^ Therefore, therapies that interfere with antibodies’ ability to bind with incompatible RBCs have become an attractive strategy. As our characterization data showed that RBC(A)‐NP and RBC(B)‐NP were able to interact with the corresponding IgM antibodies, we thus investigated if they could be leveraged to relieve the IgM‐mediated incompatible RBCs agglutination in vitro.

**Figure 2:**
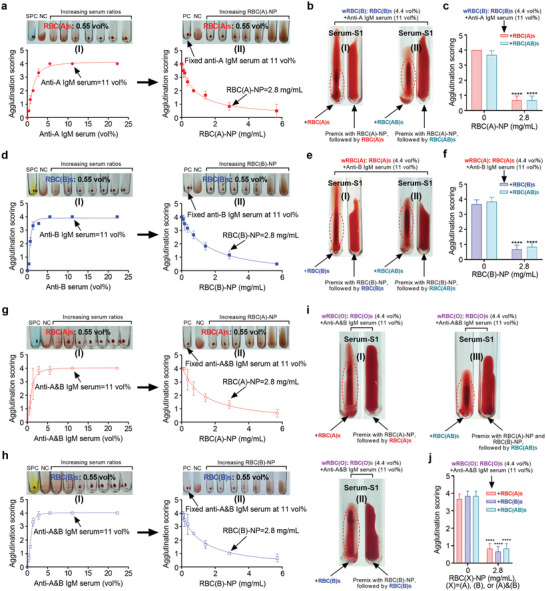
RBC‐NP blocked the corresponding IgM‐mediated incompatible blood transfusion. a) Dose‐dependent of anti‐A IgM serum (1:128 titers) agglutinated with 0.55 vol% RBC(A)s, and 11 vol% was determined to get score 4 of agglutination (I); dose‐dependent of RBC(A)‐NP against 11 vol% of anti‐A IgM serum‐mediated RBC(A)s clump, and 2.8 mg mL^−1^ was determined to drop the agglutination score from 4 to 1 (II). b) Images showing an experimental wRBC(B) pre‐incubated with 2.8 mg mL^−1^ of RBC(A)‐NP was successfully transfused with 0.55 vol% of RBC(A)s (I) or RBC(AB)s (II). The experimental wRBC(B) treated with RBC(A)s or RBC(AB)s alone were used as controls. c) Statistical analysis of agglutination scoring for (b). (*n* = 3, mean ± SD). d) Dose‐dependent of anti‐B IgM serum (1:128 titers) agglutinated with 0.55 vol% RBC(B)s, and 11 vol% was determined to reach score 4 of agglutination (I); dose‐dependent of RBC(B)‐NP against 11 vol% of anti‐B IgM serum‐mediated RBC(B)s aggregation, and 2.8 mg mL^−1^ was determined to decrease the agglutination score from 4 to 1 (II). e) Images showing an experimental wRBC(A) pre‐treated with 2.8 mg mL^−1^ of RBC(B)‐NP was compatibly transfused with 0.55 vol% of RBC(B)s (I) or RBC(AB)s (II). The experimental wRBC(A) treated with RBC(B)s or RBC(AB)s alone were used as controls. f) Statistical analysis of agglutination scoring for (e). (*n* = 3, mean ± SD). Dose‐dependent of anti‐A&B IgM serum (1:128 titers) agglutinated with 0.55 vol% g‐I) RBC(A)s or h‐I) RBC(B)s, and 11 vol% was determined to get a score 4 of agglutination; dose‐dependent of RBC(A)‐NP or RBC(B)‐NP against 11 vol% of g‐II) anti‐A&B IgM serum‐mediated RBC(A)s or h‐II) RBC(B)s clumps, and 2.8 mg mL^−1^ was determined to drop the agglutination score from 4 to 1 for both. i) Images showing an experimental wRBC(O) preincubated with 2.8 mg mL^−1^ of i‐I) RBC(A)‐NP, i‐II) RBC(B)‐NP, or i‐III) RBC(A)‐NP combined with RBC(B)‐NP were successfully transfused with 0.55 vol% of (i‐I)RBC(A)s, i‐II) RBC(B)s, and i‐III) RBC(AB)s respectively. The experimental wRBC(O) treated with RBC(A)s, RBC(B)s, or RBC(AB)s alone were used as controls. j) Statistical analysis of agglutination scoring for (i). (*n* = 3, mean ± SD). NC, negative control. SPC, standard positive control.

We first scored the agglutination grading based on the clump degree of RBCs by mixing various IgM titers with a fixed RBCs amount, where score 4 was the highest grade characterized by one big solid RBCs clump (Figure S6, Supporting Information). We then determined the minimal dosage of anti‐A IgM serum (1: 128 titer) at 11 vol% could reach a score 4 when added to 0.55 vol% RBC(A)s (**Figure** **2**a–I). Upon this dose, we next evaluated the ability of RBC(A)‐NP to protect against anti‐A IgM‐mediated RBC(A)s agglutination. By preincubating anti‐A IgM serum, increasing RBC(A)‐NP inputs offered dose‐dependent protection from IgM‐involved RBC(A)s clump. Notably, less than score 1, considered safe for blood transfusion in the clinic, was observed when preincubated with 2.8 mg mL^−1^ of RBC(A)‐NP (Figure 2a‐II). In parallel, we tested that anti‐A IgM serum (1: 128 titers) at 11 vol% followed a non‐linear curve as a neutralization function of the RBC(A)‐NP concentrations (Figure S7, Supporting Information). Based on these results, we thus speculated if wRBC(B) (contained RBC(B)s and anti‐A IgM antibodies) following the RBC(A)‐NP pretreatment could be compatible with RBC(A)s. Along with this thought, we first prepared an experimental wRBC(B) by mixing 11 vol% of anti‐A IgM serum (1: 128 titer) with 4.4 vol% RBC(B)s. Afterward, such wRBC(B) was treated with either 2.8 mg mL^−1^ of RBC(A)‐NP or an equal volume of PBS, followed by adding 0.55 vol% RBC(A)s. The results showed that pretreated wRBC(B) with RBC(A)‐NP exhibited high blood compatibility to RBC(A)s, whereas the wRBC(B) without RBC(A)‐NP pretreatment led to a big visible RBCs clump (Figure 2b‐I,c, and Video S1, Supporting Information). It concluded that RBC(A)‐NP indeed was capable of absorbing anti‐A IgM antibodies in wRBC(B), thereby diverting them away from binding with RBC(A)s. Also, by sequestering anti‐A IgM antibodies, RBC(A)‐NP could likewise allow RBC(AB)s to transfuse with wRBC(B) without observable agglutination (Figure 2b‐II,c).

A similar method to neutralizing anti‐A IgM antibodies by RBC(A)‐NP, we then confirmed that RBC(B)‐NP could be leveraged to neutralize anti‐B IgM antibodies and provided protection from anti‐B IgM‐mediated agglutination on RBC(B)s (Figure 2d‐I,II). Also, we demonstrated that by pretreating an experimental wRBC(A) (11 vol% of anti‐B IgM serum mixed with 4.4 vol% RBC(A)s) with RBC(B)‐NP at 2.8 mg mL^−1^ could noticeably block the anti‐B IgM antibodies’ ability to aggregate with RBC(B)s or RBC(AB)s (Figure 2e‐I,II, f). Furthermore, as it is hard to transfuse RBC(A)s, RBC(B)s, or RBC(AB)s to O‐type blood recipients in the clinic, we next aimed to overcome this obstacle by employing our strategy. We then verified that RBC(A)‐NP or RBC(B)‐NP could relieve anti‐A and anti‐B IgM serum (harvested from O‐type blood donors)‐mediated agglutination against RBC(A)s or RBC(B)s (Figure 2g‐I,II,h‐I,II). Similarly, it was observed that pretreatment of an experimental wRBC(O) (containing 11 vol% of anti‐A and anti‐B IgM serum and 4.4 vol% RBC(O)s) with 2.8 mg mL^−1^ of RBC(A)‐NP or RBC(B)‐NP could make blood transfusions compatible between wRBC(O) and RBC(A)s or between wRBC(O) and RBC(B)s (Figure 2i‐I,II,j). It was worth noting that neither RBC(A)‐NP nor RBC(B)‐NP alone could stop the agglutination between wRBC(O) and RBC(AB)s (Figure S8, Supporting Information). Was only the combination of RBC(A)‐NP and RBC(B)‐NP able to absorb anti‐A and anti‐B IgM antibodies simultaneously could make blood transfusion of RBC(AB)s to wRBC(O) available (Figure 2i‐III,j).

Overall, these results established that IgM antibodies regarding incompatible blood transfusions could be effectively absorbed by the corresponding RBC‐NPs, enabling the incompatible blood transfusion in the ABO blood system.

### 2.3 RBC‐NP Relieved the Destructive IgG‐Mediated Incompatible Blood Transfusion

**Figure 3:**
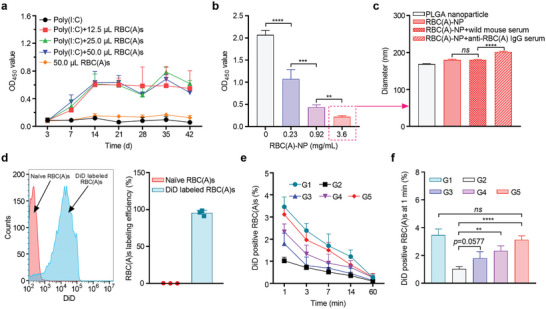
RBC‐NP protected RBCs from IgG‐mediated opsonization and clearance. a) ELISA assay detecting anti‐RBC(A) IgG antibody levels at different time points after injecting mice with various volumes of human RBC(A)s following the 100 µg of poly(I:C) prestimulation. Poly(I:C) at 100 µg only or 50 µL of RBC(A)s alone were used as controls. (*n* = 5, mean ± SD). b) ELISA assay detecting the various concentrations of RBC(A)‐NP for neutralizing the 10 vol% of anti‐RBC(A) IgG antibody serum. (*n* = 3, mean ± SD). c) Diameter changes of RBC(A)‐NP before and after incubation of anti‐RBC(A) IgG antibody serum. RBC(A)‐NP treated with wild mouse serum was used as a control. (*n* = 3, mean ± SD). d) Flow cytometry picture of DiD‐labeled RBC(A)s (left). Statistical analysis of labeling efficiency (right). Naïve RBC(A)s sample was employed as the negative control. (*n* = 3, mean ± SD). e) The remaining ratios of DiD‐labeled RBC(A)s at planned time points following the injection of various doses of RBC(A)‐NP into an established anti‐RBC(A) IgG antibody passive murine model. (*n* = 4, mean ± SD). f) The remaining ratio of DiD‐labeled RBC(A)s at the first 1 min following the injections of various doses of RBC(A)‐NP into an established anti‐RBC(A) IgG antibody passive murine model. (*n* = 4, mean ± SD). In e,f), five groups were below, G1: mice treated with PBS followed by injection of DiD‐labeled RBC(A)s; G2: mice treated with anti‐RBC(A) IgG serum followed by injection of DiD‐labeled RBC(A)s; G3: mice treated with anti‐RBC(A) IgG serum, followed by 6.3 mg kg^−1^ of RBC(A)‐NP and injection of DiD‐labeled RBC(A)s; G4: mice treated with anti‐RBC(A) IgG serum, followed by 25 mg kg^−1^ of RBC(A)‐NP and injection of DiD‐labeled RBC(A)s; G5: mice treated with anti‐RBC(A) IgG serum, followed by 100 mg kg^−1^ of RBC(A)‐NP and injection of DiD‐labeled RBC(A)s.

IgM‐coated RBCs induce intravascular hemolysis during incompatible blood transfusions, while the IgG molecules mainly govern the extravascular opsonization of RBCs by phagocytes.^[^ ^28^ ^]^ After having studied the capacity of RBC‐NP to overcome incompatible blood transfusion by neutralizing the corresponding IgM antibodies, we next investigated whether RBC‐NP could be leveraged to inhibit IgG‐mediated incompatible blood transfusion. First, the pathogenic IgG antibodies were generated through an RBC hetero‐immune model in which C57BL/6J mice received human RBC(A)s following poly(I:C) prestimulation. The reason why we used human A‐type RBC in this model is due to its high frequency in the ABO blood system compared to B or AB‐type RBCs.^[^ ^29^, ^30^ ^]^ Then, we demonstrated that the high levels of IgG antibodies specific for RBC(A)s were detected at 1 week and saturated at 2 weeks; at this time point, RBC(A)s dose at 12.5 µL or higher all have saturated for antibody titers (**Figure** **3**a). As such, mouse serum at 2 weeks after being immunized with 12.5 µL of RBC(A)s and poly(I:C) was collected and employed for subsequent experiments. Next, we verified that RBC(A)‐NP was able to decrease the anti‐RBC(A) IgG amounts in 10 vol% of mouse serum in a dose‐dependent manner, where greater than 85% neutralization efficacy was achieved at 3.6 mg mL^−1^ (Figure 3b). This result was also quantitatively correlated using 10 vol% of serum over the increasing input of RBC(A)‐NP doses (Figure S9, Supporting Information). Upon absorption of 10 vol% anti‐RBC(A) IgG by RBC(A)‐NP at 3.6 mg mL^−1^, the resultant complex (IgG‐captured RBC(A)‐NP) had a significant increase in size compared to RBC(A)‐NP alone or RBC(A)‐NP treated with wild mouse serum, again suggesting the RBC(A)‐NP indeed was able to sequester anti‐RBC(A) IgG antibodies (Figure 3c).

Following confirmation of RBC(A)‐NP functionality to absorb anti‐RBC(A) IgG antibodies in vitro, we proceeded to evaluate its in vivo performance using a passive immune murine model. Naïve mice first passively received the anti‐RBC(A) IgG serum at 50 µL intravenous, followed by injecting various doses of RBC(A)‐NP or an identical PBS volume via the tail vein. After that, fresh packed RBC(A)s labeled with DiD dye at nearly 100% labeling efficiency (Figure 3d) were injected intravenously 2 h afterward and then they were monitored at planned time points. It was shown that supplemented with RBC(A)‐NP conferred dose‐dependent protection from IgG antibody‐mediated opsonization, resulting in gradually slow clearance rates of DiD‐labeled RBC(A)s (Figure 3e). When the mice receiving the anti‐RBC(A) IgG serum followed by 100 mg kg^−1^ of RBC(A)‐NP (Figure 3e‐G5), it was observed that DiD‐labeled RBC(A)s posted a similar clearance behavior compared with mice that only receiving DiD‐labeled RBC(A)s (Figure 3e‐G1). In contrast, the DiD‐labeled RBC(A)s were significantly decreased at first 1 min when only anti‐RBC(A) IgG serum administrated without the treatment of RBC(A)‐NP (Figure 3e‐G2), compared to other groups (Figure 3f). Notably, despite without IgG antibodies, DiD labeling RBC(A)s after being injected into naïve mice would be quickly eliminated over 60 min (Figure 3e‐G1), which was probably attributable to the hetero‐blood transfusion between human RBCs and mouse circulation. Despite the availability of this passive immune murine model, there is still a limitation that the received IgG antibody serum is a mixture that not only targets A antigen but also against the other RBC membrane proteins due to the hereto‐immune pattern, which may not be very suitable for the further implications for the potential clinical applications of RBC‐NP. Thus, a new murine model is needed to study incompatible blood transfusions better in the future.

The results from this part indicated that the RBC(A)‐NP was capable of neutralizing anti‐RBC(A) IgG antibodies, thereby mitigating the IgG‐mediated RBC(A)s opsonization and clearance in mice. It was worth noting that RBC‐NP may confer a generalized strategy for overcoming incompatible blood transfusion by neutralizing incompatible blood transfusions‐targeted antibodies, not only for the ABO blood system but also for Rh, KELL, and other blood systems.

### 2.4 Assessment of eRBC‐NP Functionality before and after Lyophilization/Resuspension

RBCs have a shelf life of only up to 42 d. Accumulating studies have revealed that RBCs’ ability to transport oxygen would be weakened when they are stored hypothermically in blood banks as time elapses because the “storage lesions” upon RBCs are gradually developing and accumulating over their shelf‐life.^[^ ^31^ ^]^ Furthermore, one report showed that transfusion of packed RBCs at the end of shelf‐life would increase the risk of mortality.^[^ ^32^ ^]^ Given these situations, once the shelf‐life of RBCs is exceeded, blood banks must discard or dispose of them without further use in the clinic. As RBC‐NP is made by wrapping the RBC membrane (hemoglobin removed) onto a PLGA nanoparticle, it only harnesses the function of the RBC membrane. This is prompted that whichever “new” or “old” RBCs, as long as they maintain the incompatible blood transfusion‐related antigens that are targeted by the corresponding antibodies, it could be leveraged to neutralize those antibodies. As such, we sought to evaluate if the expired RBC membrane (derived from expired RBCs)‐coated PLGA nanoparticle (denoted eRBC‐NP) could neutralize the corresponding IgM and IgG antibodies in comparison to that of fresh RBC membrane‐camouflaged PLGA nanoparticle (denoted fRBC‐NP).

**Figure 4:**
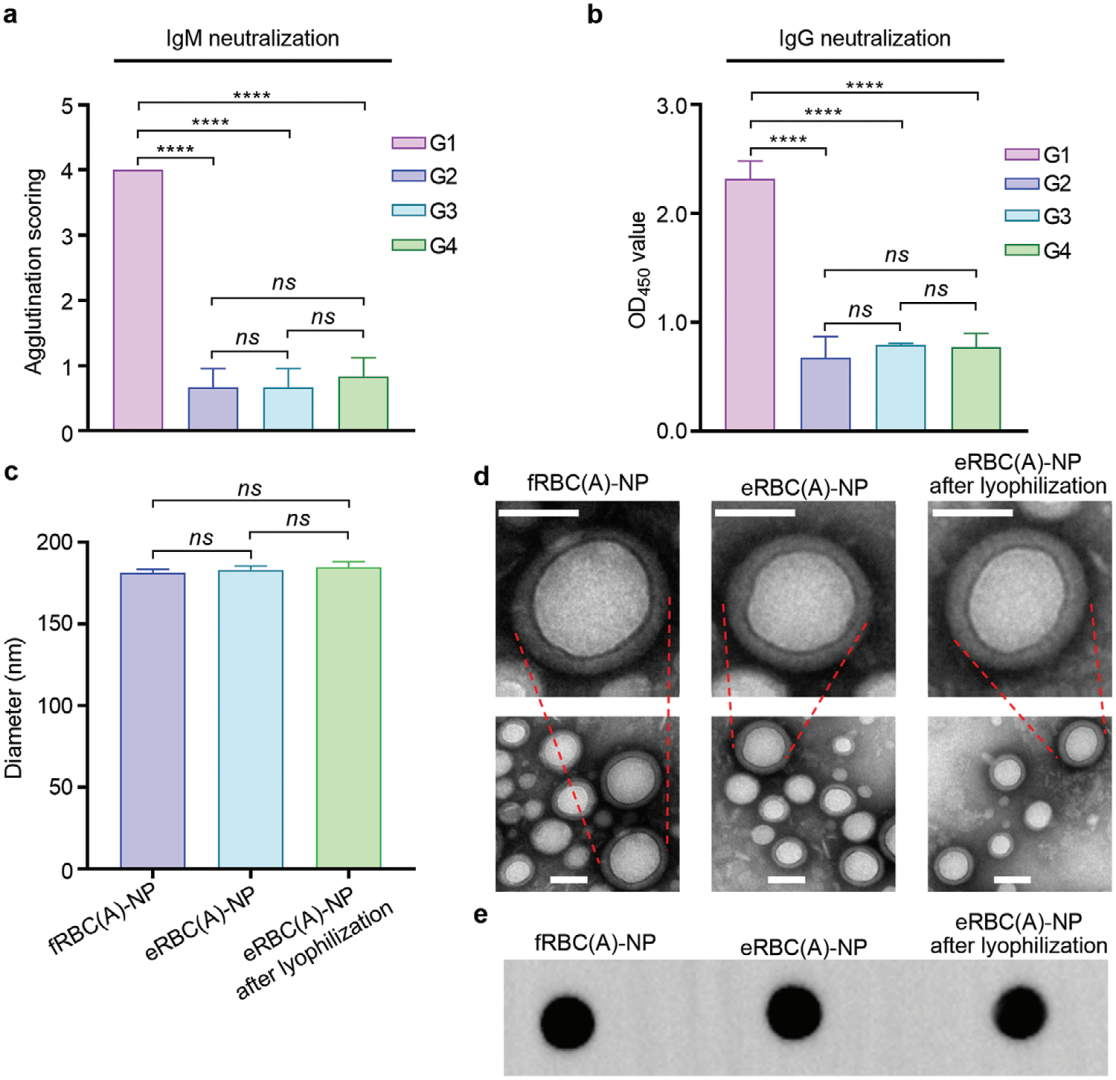
Assessment of eRBC‐NP functionality before and after lyophilization/resuspension. a) Protective effect of various RBC membrane‐based formulations against anti‐A IgM antibody‐mediated agglutination on RBC(A)s. (*n* = 3, mean ± SD). G1: anti‐A IgM serum only; G2: fRBC(A)‐NP pre‐incubated with anti‐A IgM serum; G3: eRBC(A)‐NP pre‐incubated with anti‐A IgM serum; G4: eRBC(A)‐NP following lyophilization and resuspension was pre‐incubated with anti‐A IgM serum. b) Neutralization effect of various RBC membrane‐based formulations against anti‐RBC(A) IgG serum. (*n* = 3, mean ± SD). G1: anti‐RBC(A) IgG serum alone; G2: fRBC(A)‐NP pre‐incubated with anti‐RBC(A) IgG serum; G3: eRBC(A)‐NP pre‐incubated with anti‐RBC(A) IgG serum; G4: eRBC(A)‐NP following lyophilization and resuspension was pre‐incubated with anti‐RBC(A) IgG serum. c) Hydrodynamic sizes of fRBC(A)‐NP, eRBC(A)‐NP, and eRBC(A)‐NP after treatment of lyophilization and resuspension. (*n* = 3, mean ± SD). d) Morphological images of fRBC(A)‐NP, eRBC(A)‐NP, and eRBC(A)‐NP as visualized by TEM. Bar = 50 nm. e) Antigen A profiles expressed in fRBC(A)‐NP, eRBC(A)‐NP, and eRBC(A)‐NP after treatment of lyophilization and resuspension were tested by Dot blot.

After preincubating 11 vol% of anti‐A IgM serum (1: 128), eRBC(A)‐NP at 2.8 mg mL^−1^ could drop the RBC(A)s agglutination score from 4 to 1, with comparable efficacy to fRBC(A)‐NP at an identical dose (**Figure** **4**a‐G2,G3). Also, a similar result was observed using 3.6 mg mL^−1^ of eRBC(A)‐NP or fRBC(A)‐NP, both of which could effectively absorb anti‐RBC(A) IgG (Figure 4b‐G2,G3). Having estimated the functionality of eRBC‐NP, we next evaluated its further validity after lyophilization and resuspension. We first lyophilized eRBC(A)‐NP and stored it at −80 °C for 90 d, followed by reconstitution. As a result, eRBC(A)‐NP after being treated with lyophilization and resuspension cycle not only presented no changes in hydrodynamic diameter and morphology compared to fRBC(A)‐NP (Figure 4c,d) but also did not impact its ability to neutralize the corresponding IgM and IgG antibodies (Figure 4a‐G2–G4,b‐G2–G4). The consistent functionality of eRBC(A)‐NP or lyophilized/resuspended eRBC(A)‐NP with fRBC(A)‐NP was mainly attributable to the negligible loss of A antigens or other membrane proteins during hypothermic storage in the blood bank and/or −80 °C storage after lyophilization (Figure 4e and Figure S10, Supporting Information). In addition, RBC‐NP coated with expired RBC membrane did not significantly impair the circulation time compared to that of fresh RBC membrane‐based nanoparticles (Figure S11, Supporting Information). These advantages of RBC‐NP together could largely resolve the blood supply shortage by leveraging the eRBCs; otherwise, the eRBCs would be useless and be discarded as biohazard thing. Furthermore, eRBC‐NP, after being treated by resuspension following at least 90‐day lyophilization, has been tested effective for IgM and IgG antibodies neutralization, which is expected to extend the “shelf‐life” of RBCs and facilitate the applicability of our strategy.

### 2.5 Biodistribution and Biocompatibility of IgM or IgG‐Captured RBC‐NP in a Volume‐Targeted Hemorrhagic Shock Mouse Model

**Figure 5:**
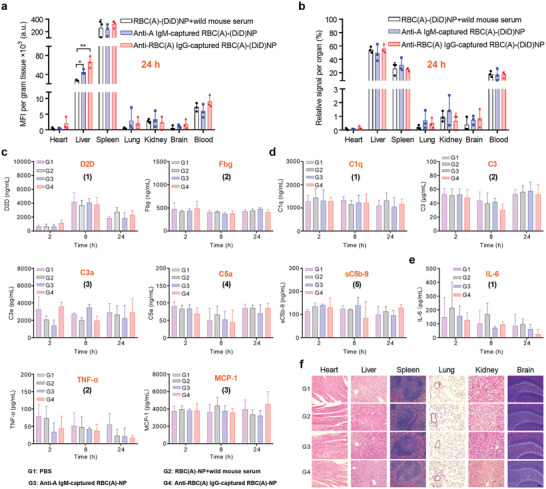
Biodistribution and biocompatibility of IgM or IgG‐sequestered RBC‐NP in a volume‐targeted hemorrhagic shock mouse model. a) Fluorescence intensity per gram of tissue or b) relative signal per organ for dissected major organs from mice 24 h after intravenous given of anti‐A IgM or anti‐RBC(A) IgG‐sequestered RBC(A)‐(DiD)NP into the hemorrhagic shock mice. Mice treated with PBS or RBC(A)‐(DiD)NP complexed with wild mouse serum were used as controls. (*n* = 3, mean ± SD). The serum concentrations regarding coagulation‐related factors including c) D2D and Fbg, complement components including d) C1q, C3, C3a, C5a, and sC5b‐9, allergic toxins including d) C3a and C5a, and inflammatory cytokines including e) IL‐6, TNF‐α, and MCP‐1, were measured by the corresponding ELISA kits on 2, 8, and 24 h post‐injection of four different treatments in the hemorrhagic shock mice. f) H&E staining of histology sections from major organs 24 h after different treatments. Scale bar = 50 µm. From c–f), four different treatments, including the hemorrhagic shock mice treated with RBC(A)‐NP complexed with anti‐A IgM or anti‐RBC(A) IgG, or RBC(A)‐NP pretreated with wild mouse serum or PBS only. (*n* = 5, mean ± SD).

Our data has shown that RBC‐NP could absorb the corresponding hemolytic IgM and IgG antibodies, and these nanoparticles are usually not used in healthy individuals. Thus, we sought to investigate the fate of RBC‐NP after absorption of antibodies in a volume‐targeted hemorrhagic shock mouse model. To track the biodistribution of anti‐A IgM or anti‐RBC(A) IgG‐sequestered RBC(A)‐NP in the hemorrhagic shock mice, the PLGA core was prelabeled with DiD dye (final formulation denoted RBC(A)‐(DiD)NP).^[^ ^25^ ^]^ At various time points including 0.25, 1, 3, and 24 h after intravenous injection, antibodies‐sequestered RBC(A)‐(DiD)NP were distributed mainly in the liver, spleen, and blood, followed by kidneys, lungs, and brain when analyzed for distribution per organ (**Figures** **5**a and S12a, Supporting Information). At the same time points, they were found to have stayed mainly in the spleen and liver when all profiles were normalized to the organ weight (Figure 5b and Figure S12b, Supporting Information). Interestingly, antibodies‐sequestered RBC(A)‐(DiD)NP, regardless of holding the IgM or IgG antibodies, exhibited a higher fluorescence level in the liver compared to that of RBC(A)‐(DiD)NP pretreated with wild mouse serum. This distribution pattern is likely attributed to the antibodies‐captured RBC(A)‐(DiD)NP that exposed the Fc fragment to the Fc receptor of phagocytes residing within the liver, facilitating the uptake of these nanoparticles. Also, this finding was verified in vitro accordingly, where preincubated RBC(A)‐(DiD)NP with the corresponding IgM or IgG antibodies could accelerate the RAW264.7 (murine macrophages) cellular uptake in a time‐dependent style, in comparison to RBC(A)‐(DiD)NP after the treatment of wild mouse serum (Figure S13, Supporting Information).

To evaluate the biosafety, the mice following the blood loss were injected with IgM or IgG‐captured RBC(A)‐NP (100 mg kg^−1^), and the relative parameters at different time points (2, 8, and 24 h) were measured. It was shown that all parameters, including D‐Dimer (D2D) and Fbg for disseminated intravascular coagulopathy (DIC) (Figure 5c‐1,2), C1q, C3, C3a, C5a, and sC5b‐9 for complement activation (Figure 5d‐1–5), C3a and C5a for anaphylatoxins (Figure 5d‐3,4), and IL‐6, tumor necrosis factor‐alpha (TNF‐α), and monocyte chemoattractant protein‐1(MCP‐1) for inflammation (Figure 5e‐1–3), were consistent with those from the hemorrhagic shock mice receiving PBS only. At 24 h, major organs from these mice were collected and sectioned, and the histological evaluation of these tissue samples was performed after being stained with hematoxylin and eosin (HE). It was shown that the major organs, including the heart, liver, spleen, lungs, kidneys, and brain revealed no significant irregularities (Figure 5f). In addition, IgM or IgG‐captured RBC(A)‐NP at a higher dose of 200 mg kg^−1^ also exhibited great biocompatibility in the volume‐targeted hemorrhagic shock mouse model (Figure S14, Supporting Information). These results together indicated a general safety and biocompatibility of IgM or IgG‐sequestered RBC‐NP in the hemorrhagic shock mice.

## 3 Conclusions

**Figure 6:**
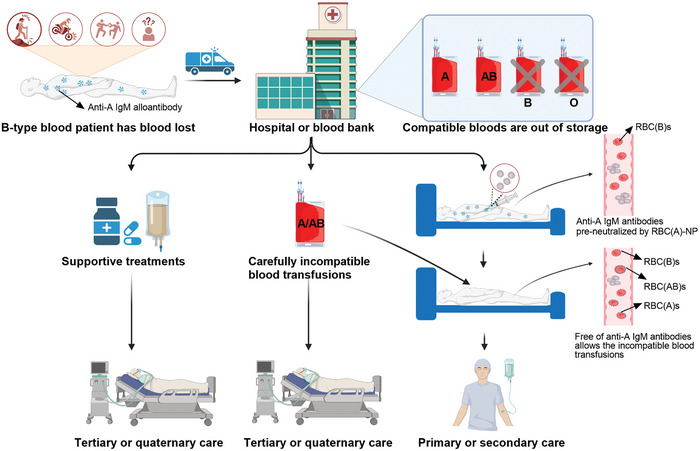
RBC‐NP enables incompatible blood transfusions in an emergency. By neutralization of anti‐A IgM antibodies in B‐type blood recipients in an emergency where there is a lack of compatible RBC resources in hospitals or blood banks, RBC(A)‐NP enables the incompatible transfusion of donor RBCs, including RBC(A)s or RBC(AB)s. Also, this strategy could be employed to compatibly transfuse RBC(B)s or RBC(AB)s into A‐type blood recipients or transfuse RBC(A)s, RBC(B)s, or RBC(AB)s into O‐type blood recipients when incompatible blood transfusion‐related IgM antibodies could be first neutralized by the corresponding RBC‐NP. Similarly, RBC‐NP could be leveraged to block the destructive IgG‐induced RBC opsonization and clearance. This strategy can save lives or improve health in emergencies; otherwise, patients might be subjected to tertiary or quaternary care after supportive treatments or carefully incompatible blood transfusions. Overall, this strategy is expected to be applied in different blood group systems, including ABO, Rh, and others in emergencies, and lessens the tight blood supply worldwide.

In conclusion, we have developed a simple and effective RBC‐NP nanoplatform to enable incompatible blood transfusions. With different blood types of RBC membrane coatings, RBC‐NPs holding the parent RBCs’ alloantigens were capable of pre‐neutralizing the corresponding blood transfusion‐related antibodies, including IgM and IgG in recipients, and diverting the antibodies away from binding at incompatible donor RBCs, thereby overcoming the incompatible blood transfusions. In this study, a successful blood transfusion was achieved when RBC(A)s or RBC(AB)s were added to wRBC(B) following the RBC(A)‐NP pretreatment for anti‐A IgM antibody neutralization. Similarly, pretreated wRBC(A)s or wRBC(O)s with RBC(B)‐NP or a combination of RBC(B)‐NP and RBC(A)‐NP could make the blood transfusions available between wRBC(A)s and RBC(B)s or RBC(AB)s, or between wRBC(O)s and RBC(A)s, RBC(B)s, or RBC(AB)s. Also, anti‐RBC(A) IgG‐induced RBC(A)s opsonization in murine circulation was relieved through pretreating mice with RBC(A)‐NP, and this effect could last at least 48 h (Figure S15, Supporting Information). Furthermore, the fate of eRBCs would be rewritten as they could be leveraged to fabricate eRBC‐NP that has been verified via a similar effect at neutralizing the corresponding IgM or IgG antibodies compared to fRBC‐NP. Impressively, IgM or IgG‐captured RBC‐NP did not further challenge the state of the body in the hemorrhage shock mice. Overall, the strategy to enable the incompatible blood transfusion in an urgent situation is highly desirable (e.g., a life‐threatening recipient is a B‐type blood, but the hospital or blood bank currently has A/AB‐type RBCs only, how should the clinician decide? (**Figure** **6**)), and we envision that this type of incompatible antibodies‐neutralizing approach may be applied to resolve the incompatible blood transfusions for ABO or other blood group systems in the emergencies in the near future.

## 4 Experimental Section

### Materials

Bicinchoninic acid (BCA) kit was purchased from Thermo Fisher (America). DiD, DiO, enhanced chemiluminescence (ECL) western blot substrate, radioimmunoprecipitation assay (RIPA) lysis buffer and Dulbecco's modified Eagle medium (DMEM) were purchased from Meilunbio (China). 4′,6‐Diamidino‐2‐phenylindole (DAPI) and bovine serum albumin (BSA) were purchased from Sigma‐Aldrich (America). ELISA coating buffer (5×), 3,3′,5,5′ tetramethylbenzidine (TMB) substrate solution, and stop solution were obtained from Biolegend (America). Carboxyl group‐terminated 50:50 PLGA at 0.67 dL g^−1^ was purchased from Lactel Absorbable Polymers (America). Omni‐Easy Protein Sample Loading Buffer (5×) was purchased from Epizyme (China). Coomassie Blue was purchased from Solarbio (China). Blood group A antigen goat anti‐mouse antibody (Z2A) and blood group B antigen goat anti‐mouse antibody (Z2H) were purchased from Santa Cruz (America). Horseradish peroxidase (HRP)‐conjugated IgM µ Chain rabbit anti‐goat secondary antibody was purchased from Boster (China). Anti‐A and anti‐B blood grouping reagents were purchased from SHPBC (China). Fetal bovine serum (FBS) and penicillin–streptomycin were purchased from Gibco. High‐molecular‐weight poly(I:C) and HRP‐conjugated IgG Fc secondary antibody were purchased from InvivoGen (America). Anticoagulant preservative solution was purchased from KWS (China). D‐Dimer (D2D), Fbg, C1q, C3, C3a, C5a, sC5b‐9, IL‐6, TNF‐α, MCP‐1, and IL‐6 ELISA kit were purchased from Elabscience (China).

### Derivation of Human RBC Membrane

Different blood types (including A, B, O, and AB) of human whole blood were collected and tested virus‐free at the Department of Blood Transfusion, The Second Affiliated Hospital of Wenzhou Medical University. Then, the RBC membrane was derived in accordance with our previous works.^[^ ^23^, ^24^, ^25^ ^]^ Briefly, whole blood was first centrifuged at 1000 *g* for 5 min, and the resulting plasma at the top layer and bottom packed RBCs pellet was harvested. After that, the packed RBCs were suspended in water and placed in the ice bath for 10 min, then adjusted to 1× PBS. Afterward, the sample was centrifuged at 13 000 *g* for 5 min, followed by hemoglobin removal in supernatant. This hypotonic treatment was repeated until the supernatant was out of hemoglobin. The final pelleted RBC membrane was resuspended in water, and a BCA assay determined membrane protein concentration according to the manufacturer's instructions. This study was approved by the Ethics Committee of the Second Affiliated Hospital of Wenzhou Medical University (2022‐K‐317‐01).

### Preparation of RBC(A)‐NP and RBC(B)‐NP

PLGA polymeric nanoparticles were first prepared with a nanoprecipitation method, where 2 mL of water was added to 1 mL of PLGA (dissolved in acetone, 20 mg mL^−1^). The mixture was stirred in a vacuum chamber to evaporate acetone completely. Then, RBC‐NP was prepared using a bath ultrasonicator (Fisherbrand, FB15051). Various weights of RBC membrane (0.25, 0.5, and 1 mg) were mixed with 1 mg of PLGA cores in a final volume of 1 mL, followed by bath ultrasonication at 100 W for 2 min. Following the sonication, the optimal ratio of RBC membrane to PLGA nanoparticle at 0.5: 1 (w/w) was determined by dynamic laser scattering (DLS) measurement using a Zetasizer Nano ZS (Malvern).

### Characterization of RBC(A)‐NP and RBC(B)‐NP

At the RBC membrane/PLGA core ratio of 0.5, RBC(A)‐NP and RBC(B)‐NP coated with RBC(A) membrane and RBC(B) membrane were synthesized respectively, and DLS measured their hydrodynamic diameters and zeta potentials. Meanwhile, DLS monitored the colloidal stability of RBC(A)‐NP and RBC(B)‐NP in 10% FBS at days 1, 3, 5, and 7 after synthesis. PLGA nanoparticle only was used as a control.

The morphologies of RBC(A)‐NP and RBC(B)‐NP were visualized by TEM. One drop of RBC(A)‐NP or RBC(B)‐NP at 0.5 mg mL^−1^ was added onto the holey carbon‐coated Cu grids, followed by staining with 1% uranyl acetate. After drying, images were photographed with a TEM device (JEM‐2010).

Protein compositions of the RBC(A) membrane, RBC(A)‐NP, RBC(B) membrane, and RBC(B)‐NP were characterized by SDS‐PAGE. Briefly, all four samples at an equivalent protein concentration of 2 mg mL^−1^ were mixed with 5× loading buffer, followed by heating at 90 °C for 10 min. The heated samples were added to a 10‐well Blot 10% SDS‐PAGE gel and run at 150 V for 50 min. Following electrophoresis, the gel was stained with Coomassie Blue and photographed.

The blood transfusion‐related A or B antigens on the respective RBC(A)‐NP or RBC(B)‐NP was characterized by a Dot blot. Briefly, 2 µL of samples containing RBC(A) membrane, RBC(A)‐NP, RBC(B) membrane, and RBC(B)‐NP at an equivalent concentration of 1 mg mL^−1^ were spotted onto the PVDF membranes following the methanol activation for 2 min. After drying, the PVDF membranes were blocked with 5% skim milk for 30 min, followed by washing with 1× TBST (containing 0.1% Tween‐20) thricely. After that, the PVDF membranes were incubated with anti‐A antigen (1: 800 dilution) or anti‐B antigen (1: 800 dilution) primary antibodies for 2 h at room temperature (RT), followed by washing unbound antibodies away using 1× TBST for three times. Then, the primary antibody‐coated PVDF membranes were incubated with HRP‐conjugated IgM µ Chain secondary antibody (1:5000 dilution) for another 1 h at RT. The resultant membranes were washed with 1 × TBST three times. The blot signals in membranes were visualized by an ECL detection kit and were photographed by a ChemiDoc CRS imaging system (Bio‐Rad, USA). The gray density in membranes was quantified with Image J software.

### Interactions between RBC‐NP and the Corresponding IgM Alloantibodies

SPR approach was employed to test the interactions between RBC membranes and the corresponding IgM alloantibodies. Briefly, gold chips were incubated in 1 × 10^−3^ m n‐octadecyl mercaptan (dissolved in ethanol) overnight at 4 °C. After washing with PBS, chips were installed into an SPR device (Sensby T210), followed by injecting the RBC(A) membrane or RBC(B) membrane (40 µg mL^−1^, 0.8 mL) and incubated for 3 h to immobilize the membranes onto chips. After removing unbound membranes, the chips were blocked by BSA (1 mg mL^−1^) for 1 h at RT, followed by washing with PBS. Afterward, various titers of anti‐A or anti‐B IgM serums (containing 1: 32, 1: 64, and 1: 128) were injected and flowed over the chips for 15 min at RT. Before injecting a new serum, the system was first equilibrated using PBS. The data were analyzed with the state model of SPR T210 evaluation software (Sensby, China).

Next, the interactions between RBC‐NP and the corresponding IgM alloantibodies were further investigated by DLS measurements. First, RBC(A)‐NP or RBC(B)‐NP at 1 mg mL^−1^ was mixed with respective 20 vol% of anti‐A or anti‐B IgM serums (1: 128 titers) for 30 min at RT. After the removal of unattached antibodies through centrifugation at 13 000 *g* for 10 min, the pellets were resuspended by bath ultrasonication, and their hydrodynamic sizes were measured by DLS. RBC(A)‐NP and RBC(B)‐NP alone or incubated with 20 vol% of wild mouse serums were used as controls. PLGA nanoparticle was used as another control.

### Protection of RBCs from the Corresponding IgM Antibody‐Mediated Agglutination Using RBC‐NP

Firstly, the minimal ratios of anti‐A (1: 128 titers) or anti‐B IgM serums (1: 128 titers) to reach score 4 of agglutination in 0.55 vol% of RBC(A)s or RBC(B)s were determined. Briefly, various dilutions of anti‐A or anti‐B IgM serums (0.34, 0.69, 1.4, 2.8, 5.5, 11, and 22 vol%) were respectively added to 0.55 vol% RBC(A)s or RBC(B)s in a fixed volume of 450 µL. After incubation, the samples were centrifuged at 2000 rpm for 1 min and shaken for agglutination tests. Under these conditions, 11 vol% of anti‐A (1:128 titers) or anti‐B IgM serums (1:128 titers) were determined to completely agglutinate the corresponding 0.55 vol% RBC(A)s or RBC(B)s (score 4). Then, to test the anti‐agglutination effect of RBC‐NP, various concentrations of RBC(A)‐NP or RBC(B)‐NP (0.088, 0.18, 0.36, 0.7, 1.4, 2.8, and 5.6 mg mL^−1^) were premixed with 11 vol% of anti‐A (1: 128 titers) or anti‐B IgM serums (1: 128 titers) and incubated for 20 min at RT, followed by the addition of 50 µL of 5 vol% RBC(A)s or RBC(B)s to a fixed volume of 450 µL and incubation for another 20 min at RT. Afterward, the mixtures were centrifuged at 2000 rpm for 1 min, and the agglutination scorings were scored. In the study, RBC(A)‐NP or RBC(B)‐NP at 2.8 mg mL^−1^ were selected to decrease the agglutination scoring from 4 to 1. Similarly, 11 vol% of serum collected from wRBC(O) carrying anti‐A&B IgM serum (1: 128 titers for both anti‐A and anti‐B IgM antibodies) was also determined for agglutinating 0.55 vol% of RBC(A)s or RBC(B)s completely (score 4); while 2.8 mg mL^−1^ of RBC(A)‐NP or RBC(B)‐NP were found to prevent the 11 vol% of anti‐A&B IgM serum‐mediated RBC(A)s or RBC(B)s agglutination.

To test the RBC‐NP's ability to overcome incompatible blood transfusions, various experimental whole blood types, including wRBC(A), wRBC(B), and wRBC(O), were established first. Briefly, 50 µL 40 vol% of RBC(A)s, RBC(B)s, and RBC(O)s (4.4 vol% to a final volume of 450 µL) were respectively mixed with 11 vol% of anti‐B, anti‐A, and anti‐A&B IgM antibodies (1: 128 titers). Then, the experimental wRBC(A) carrying RBC(A)s and anti‐B IgM alloantibodies was pre‐incubated with 2.8 mg mL^−1^ RBC(B)‐NP for 20 min at RT, followed by adding 50 µL 5 vol% of RBC(B)s or RBC(AB)s to a final volume of 450 µL and incubation for additional 20 min at RT. Following the incubation, the mixtures were centrifuged at 2000 rpm for 1 min, followed by shaking and imaging for agglutination analysis. Similar to wRBC(A), the experimental wRBC(B) was pre‐treated with RBC(A)‐NP of 2.8 mg mL^−1^ and then mixed with 50 µL 5 vol% of RBC(A)s or RBC(AB)s upon the same conditions. In parallel, the experimental wRBC(O) containing both anti‐A IgM and anti‐B IgM was premixed with either RBC(A)‐NP or RBC(B)‐NP at 2.8 mg mL^−1^. After incubation at RT for 20 min, the mixtures were mixed with 50 µL 5 vol% of RBC(A)s or RBC(B)s to a final volume at 450 µL and incubated for 20 min under RT. The resultant samples were then centrifuged at 2000 rpm for 1 min, followed by shaking and photographed for an agglutination test. On the other hand, to simultaneously neutralize anti‐A&B IgM antibodies in wRBC(O) for RBC(AB)s transfusion, the experimental wRBC(O) was premixed with RBC(A)‐NP and RBC(B)‐NP at a respective concentration of 2.8 mg mL^−1^ and incubated for 20 min at RT. Following incubation, the samples were subjected to the addition of 50 µL 5 vol% RBC(AB)s to a final volume of 450 µL and incubated for another 20 min at RT. Afterward, the mixtures were centrifuged at 2000 rpm for 1 min, and the agglutination scorings were determined after slight shaking. The groups treated with donor RBCs alone (including A, B, or AB) were used as the respective controls.

### Protection of RBCs from the Corresponding IgG Antibody‐Mediated Opsonization and Destruction Using RBC‐NP

All animal experiments were performed under the guidelines of the Laboratory Animal Ethics Committee of Wenzhou Medical University & Laboratory Animal Centre of Wenzhou Medical University in Wenzhou, China (wydw2022‐0250). Healthy C57BL/6J mice (18–20 g) were used for all animal studies. Mice were pretreated with 100 µg of poly(I:C) per mouse by intraperitoneal, followed by intravenously injecting various volumes of human RBC(A)s (12.5, 25, and 50 µL) 3 h afterward. Then, whole blood was collected at set time points (3, 7, 14, 21, 28, 35, and 42 d) through the submandibular puncture and centrifuged at 1000 *g* for 10 min to obtain serum for anti‐RBC(A) IgG antibody quantification using ELISA. Briefly, 96‐well plates were precoated with RBC(A) membrane (20 µg mL^−1^, 100 µL per well) using 5× ELISA coating buffer at 4 °C overnight. After removing the free membrane, the wells were blocked with 1% BSA for 1 h at RT. Then, 100 µL of serums (1:100 dilution) were added and incubated for 1 h at RT. After washing with PBS, the HRP‐conjugated secondary antibody was supplemented and incubated for another 1 h at RT. After that, the wells were washed with PBS three times and developed with 100 µL per well of TMB substrate for 15 min. Then, the reaction was terminated by adding 100 µL of stop solution. Finally, the absorbance at 450 nm was measured with a plate reader (SpectraMax I3 MD USA). Upon these processes, the serums harvested from mice received 12.5 µL of human RBC(A)s following the prestimulation of 100 µg poly(I:C) at 14 d was selected and pooled for the subsequent experiments. Serums collected from mice that received either 100 µg poly(I:C) or 50 µL of human RBC(A)s only were used as controls.

To test the ability of RBC(A)‐NP to neutralize anti‐RBC(A) IgG antibody, the 96‐well plates were coated with RBC(A) membrane (20 µg mL^−1^, 100 µL per well) using 5× ELISA coating buffer at 4 °C overnight. Meanwhile, the 50 µL of pooled serums (10 vol% to final volume of 500 µL) were pre‐incubated with different concentrations of RBC(A)‐NP (0.23, 0.92, and 3.6 mg mL^−1^) at a fixed volume of 500 µL for 2 h at RT, followed by centrifuging at 13 000 *g* for 10 min. After that, 100 µL of supernatants were added to RBC(A) membrane‐coated 96‐well plates and incubated for another 1 h at RT. Afterward, the wells were treated according to the ELISA process described above. In parallel, the interactions between RBC(A)‐NP and anti‐RBC(A) IgG antibodies were further investigated by DLS measurements. First, RBC(A)‐NP at 3.6 mg mL^−1^ was mixed with 50 µL of pooled serums at 500 µL volume for 1 h at RT. After removing the free antibodies through centrifugation at 13 000 *g* for 10 min, the nanoparticle pellets were resuspended by bath ultrasonication, and their hydrodynamic sizes were measured by DLS. RBC(A)‐NP alone or incubated with 50 µL of wild mouse serum were used as controls. PLGA nanoparticle was used as another control.

Next, to test the capacity of RBC(A)‐NP to mitigate the anti‐RBC(A) IgG‐mediated RBC(A)s opsonization and clearance in mouse circulation, the RBC(A)s were prelabeled with DiD dye. Briefly, 50 µL of DiD (1 mg mL^−1^, dissolved in DMSO) was mixed well with 5 vol% of RBC(A)s dispersed in 10 mL PBS. After co‐incubating for 30 min at 37 °C, the RBCs were washed with PBS and then subjected to flow cytometry (Cytoflex, Beckman) for labeling efficiency test. Meanwhile, a murine passive immunization model was established by intravenously injecting 50 µL of pooled serum (containing anti‐RBC(A) IgG antibodies) into naïve mice, followed by immediate injection of various doses of RBC(A)‐NP (6.3, 25, and 100 mg kg^−1^) by intravenous. At 2 h after nanoparticles were given, the mice were injected with 75 µL of DiD‐labeled RBC(A)s, and the whole blood was collected at planned time points (1, 3, 7, 14, and 60 min). After centrifugation of whole blood at 1000 *g* for 10 min, the pelleted RBCs were resuspended in PBS and subjected to flow cytometry (Cytoflex, Beckman) for DiD‐positive RBC(A)s analysis. This study was approved by the Ethics Committee of the Second Affiliated Hospital of Wenzhou Medical University (2022‐K‐317‐01).

### The Ability of eRBC‐NP to Neutralize Antibodies before and after Lyophilization/Resuspension

Expired RBC(A)s (eRBC(A)s, > 42 days stored hypothermically) were collected first. Then, the RBC membranes derived from fresh RBC(A)s (fRBC(A)s) and eRBC(A)s were employed for making fRBC(A)‐NP and eRBC(A)‐NP according to a pre‐determined ratio of PLGA nanoparticle to RBC membrane. Furthermore, eRBC(A)‐NP was subjected to lyophilization treatment, stored at −80 °C for 90 d, and then reconstituted with PBS. Afterward, fRBC(A)‐NP and eRBC(A)‐NP before or after lyophilization/resuspension were used to test their anti‐antibodies effects. For IgM neutralization, all three types of RBC(A)‐NPs at 2.8 mg mL^−1^ were premixed with 11 vol% of anti‐A (1: 128 titers) and incubated for 20 min at RT, followed by the addition of 50 µL of 5 vol% RBC(A)s to a fixed volume of 450 µL and incubation for another 20 min at RT. Afterward, the mixtures were centrifuged at 2000 rpm for 1 min, and the agglutination scorings were scored. For IgG neutralization, all three types of RBC(A)‐NPs at 3.6 mg mL^−1^ were premixed with 50 µL of pooled anti‐RBC(A) IgG serums at a fixed volume of 500 µL for 2 h at RT, followed by centrifuging at 13 000 *g* for 10 min. After that, 100 µL of supernatants were added to RBC(A) membrane‐coated 96‐well plates and incubated for another 1 h at RT. Afterward, the wells were treated according to the ELISA process described above.

To test the morphology changes of fRBC(A)‐NP and eRBC(A)‐NP before and after lyophilization, all three types of RBC(A)‐NPs were photographed with a TEM device (JEM‐2010) after treatment of the same process as described in the section of “Preparation and characterization of RBC(A)‐NP and RBC(B)‐NP” in Experimental Section. Likewise, the blood transfusion‐related A antigen on the fRBC(A)‐NP and eRBC(A)‐NP before and after lyophilization were characterized by Dot blot as well as following the previous section of “Preparation and characterization of RBC(A)‐NP and RBC(B)‐NP” in Experimental Section.

### Biodistribution and Biocompatibility of IgM or IgG‐Sequestered RBC‐NP in a Volume‐Targeted Hemorrhagic Shock Mice

DiD dye dissolved in acetone was first premixed with PLGA acetone solution at a 2 wt% ratio, and the DiD‐labeled PLGA nanoparticle ((DiD)NP) was fabricated by the process same to that for PLGA synthesizing. Afterward, RBC(A)‐(DiD)NP was manufactured by sonicating RBC(A) membrane with polymeric (DiD)NP for 2 min using a bath ultrasonicator. Then, 2.8 mg mL^−1^ of RBC(A)‐(DiD)NP mixed with 11 vol% of anti‐A IgM serum (1: 128) or 3.6 mg mL^−1^ of RBC(A)‐(DiD)NP combined with 10 vol% of anti‐RBC(A) IgG serum (pooled) were incubated for 1 h, and the resultant mixtures were subjected to centrifugation of 13 000 *g* for 10 min. After centrifugation, the pelleted nanoparticles were redispersed in PBS. Meanwhile, a volume‐targeted hemorrhagic shock murine model was constructed by taking 300 µL of blood from the C57/B6 mouse via submandibular puncture. At 15 min following the blood loss, mice were injected with anti‐A IgM or anti‐RBC(A) IgG‐captured RBC(A)‐(DiD)NP at 50 mg kg^−1^ intravenously. At 0.25, 1, 3, and 24 h after giving particles, mice were euthanized, and major organs (including heart, liver, spleen, lungs, kidneys, and brain) and whole blood were harvested. Whole blood at 30 µL was added to 70 µL of water, and the organs were weighed and homogenized in PBS (1 g tissue added into 1 mL PBS) for fluorescence measurements at an excitation/emission of 630/670 nm using a plate reader. Mice treated with PBS served as the negative control.

In parallel, the biocompatibility of anti‐A IgM or anti‐RBC(A) IgG‐captured RBC(A)‐(DiD)NP in hemorrhagic shock mice was evaluated. Briefly, a volume‐targeted hemorrhagic shock murine model was first constructed by taking 300 µL of blood from the C57/B6 mouse via submandibular puncture. At 15 min following the blood loss, the mice were intravenous injected with anti‐A IgM or anti‐RBC(A) IgG‐captured RBC(A)‐NP (100 mg kg^−1^). At 2, 8, and 24 h after treatments, the whole blood samples were collected through the submandibular puncture and then were centrifuged at 1000 *g* for 5 min. Following the centrifugation, the serums were collected for different parameters measurements using the corresponding ELISA kits according to the manufacturer's instructions, including D‐Dimer (D2D), Fbg, C1q, C3, C3a, C5a, sC5b‐9, IL‐6, TNF‐α, and MCP‐1. The hemorrhagic shock mice treated with PBS or RBC(A)‐NP pretreated with wild mouse serum were used as controls. At 24 h after particle injection, major organs (heart, liver, spleen, lungs, and kidneys) were collected from all mice and were assayed for histological analysis after H&E staining.

### Statistical Analysis

The data were presented as the mean ± SD and were representative of at least three independent experiments. Statistical significance was determined by a two‐tailed unpaired Student's t‐test. Comparisons between groups were analyzed with a one‐way analysis of variance (ANOVA). Graphs were plotted, and appropriate statistical analyses were carried out using GraphPad Prism 8.0 (^*^ *p* < 0.1, ^**^ *p* < 0.01, ^***^ *p* < 0.001, and ^****^ *p* < 0.0001).

## Conflict of Interest

The authors declare no conflict of interest.

## Author Contributions

X.Y., M.C., and C.W. contributed equally to this work. M.C., J.L., W.Z., and Y.C. conceived and designed the experiments; X.Y., M.C., C.W., D.Z.G., F.J., Y.X., D.T., Q.Y., L.L., and G.S. performed all the experiments. X.Z., X.L., L.Y., L.W., Y.Z., and F.W., and B.W. participated in the guidance of the work. X.L., L.Y., L.W., F.W., J.L., W.Z., and Y.C. analyzed the data. The manuscript was written by M.C. and Y.C. All authors discussed the results and reviewed the manuscript.

## Supporting information

Supporting Information

Supporting Video 1

## Data Availability

The data that support the findings of this study are available in the supplementary material of this article.
