# Supplementary material for: Red Blood Cell Membrane‐Coated Nanoparticles Enable Incompatible Blood Transfusions
Source: Adv Sci (Weinh). 2024 Jun 5;11(29):2310230. doi: 10.1002/advs.202310230 (PMC11304279; doi:10.1002/advs.202310230)
Supplement: Supplementary file 1 — Supporting Information [file ADVS-11-2310230-s001.docx]

**Red blood cell membrane-coated nanoparticles enable incompatible blood transfusions**

Xuewei Yang^1#^, Mengchun Chen^2,4#^, Cuiye Weng^3#^, Deli Zhuge^4^, Fangsi Jin^5^, Yingnan Xiao^4^, Dongyan Tian^1^, Qingqing Yin^1^, Li Li^1^, Xufei Zhang^7^, Genghe Shi^1,7^, Xiaosheng Lu^1^, Linzhi Yan^1^, Ledan Wang^1^, Bin Wen^7^, Yingzheng Zhao^4^, Jiajin Lin^5*^, Fang Wang^1*^, Weixi Zhang^3*^, Yijie Chen^1,4,6,7*^

1. Department of Obstetrics and Gynecology, The Second Affiliated Hospital of Wenzhou Medical University, Wenzhou, 325027, China

2. Department of Pharmacy, The Second Affiliated Hospital of Wenzhou Medical University, Wenzhou, 325027, China

3. Department of Pediatric Allergy and Immunology, The Second Affiliated Hospital of Wenzhou Medical University, Wenzhou, 325027, China

4. Department of Pharmaceutics, School of Pharmaceutical Sciences of Wenzhou Medical University, Wenzhou, 325035, China

5. Department of Blood Transfusion, The Second Affiliated Hospital of Wenzhou Medical University, Wenzhou, 325027, China

6. Cixi Biomedical Research Institute, Wenzhou Medical University, Ningbo, 315302, China.

7. Wenzhou Medical University, Wenzhou, 325027, China

^#^These authors contributed equally to this work.

^*^Corresponding authors.

*E-mail addresses*: ljj88879099@126.com (Jiajin Lin), wf2060@163.com (Fang Wang), zhangweixi112@163.com (Weixi Zhang), chenyijie@wmu.edu.cn (Yijie Chen)

**Figure S1. PDI values of all sized RBC-NPs in PBS**


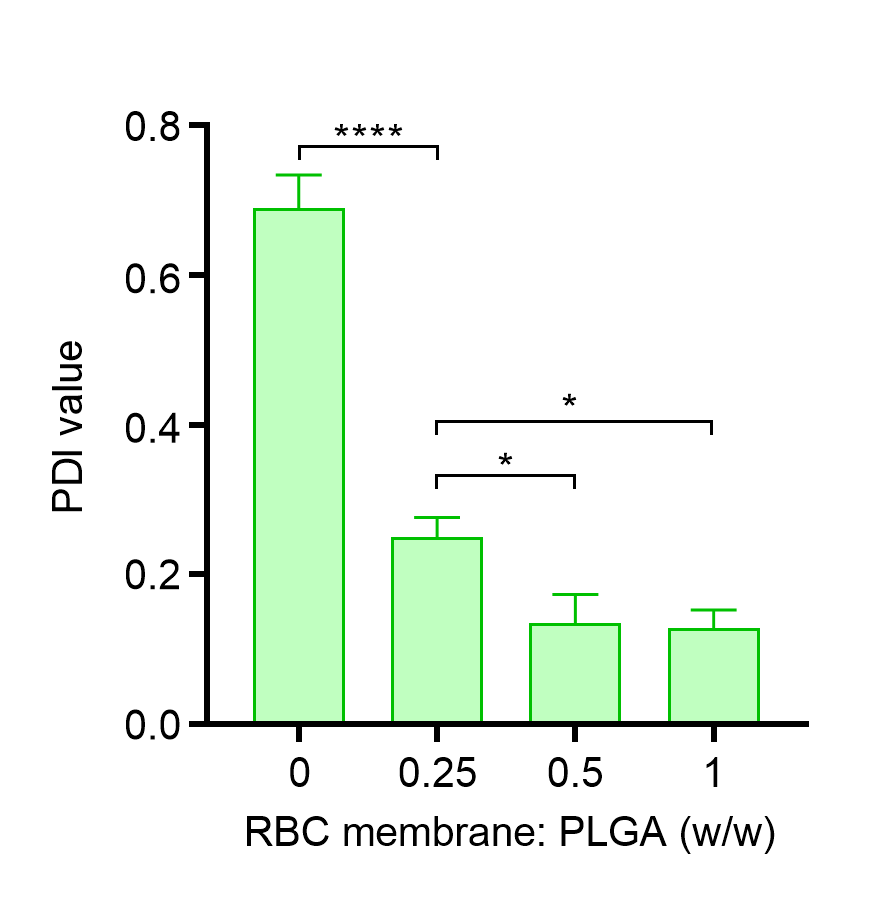


**Figure S1.** PDI values of RBC-NP formulated with various RBC membrane to PLGA nanoparticles after adjusting to 1× PBS. (n = 3, mean ± sd).

**Figure S2. TEM image of PLGA nanoparticle**


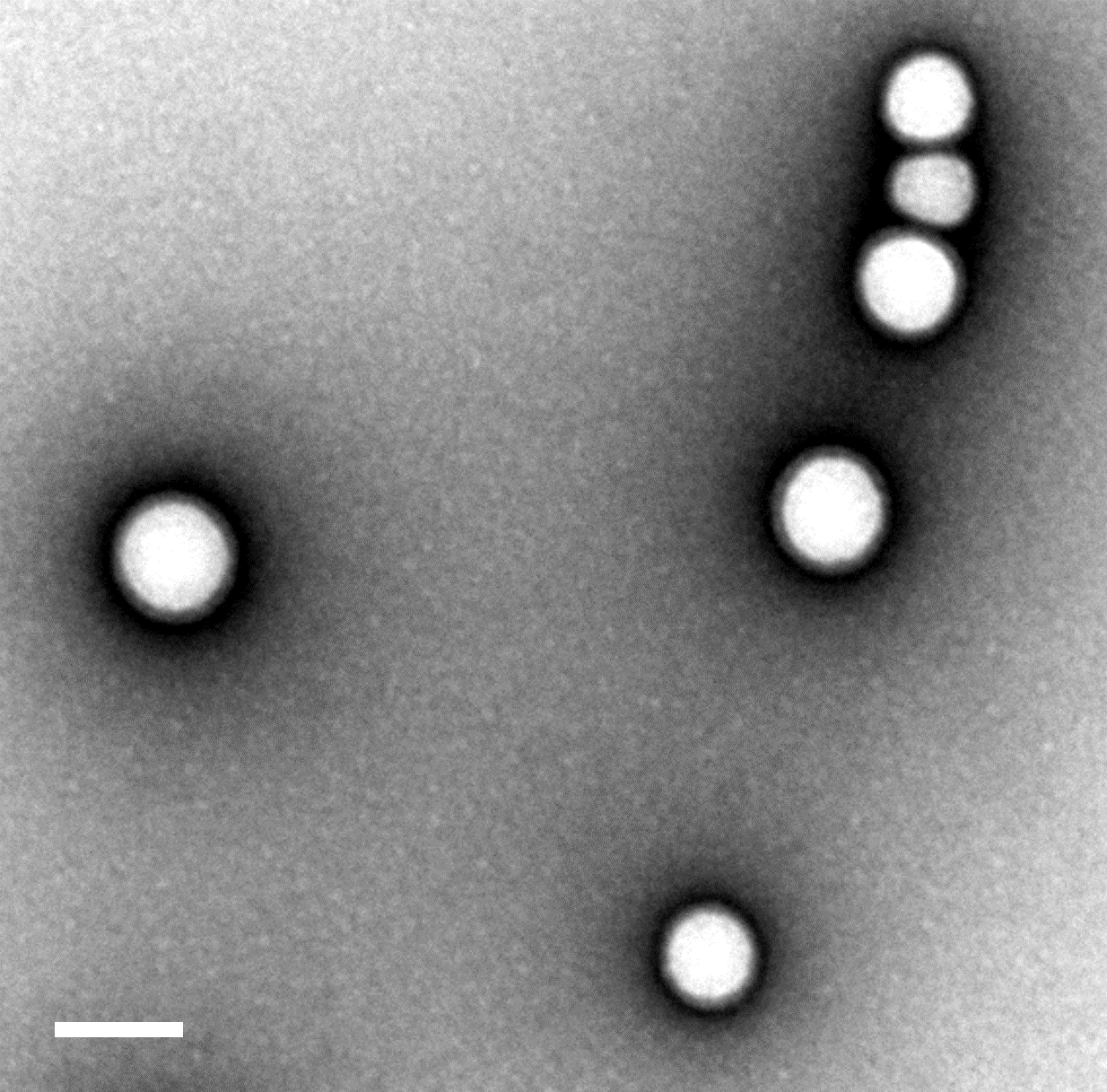


**Figure S2.** Morphology of PLGA nanoparticle as visualized by TEM. Bar = 200 nm.

**Figure S3. Fluorescence images of RBC-NP with dual-dye-labeling**

To confirm the coating of PLGA nanoparticle by the RBC membrane, RBC(A) or RBC(B) membranes stained with 0.2 wt% of DiD were sonicated with DiO-labeled PLGA cores at the optimized 0.5:1 ratio (wt%). These dual-dye decorated particles were fixed in optimal cutting temperature (OCT) compound and then placed onto polylysine-coated slides, followed by examination fluorescence colocalization using a confocal microscope (Leica TCS sp8).


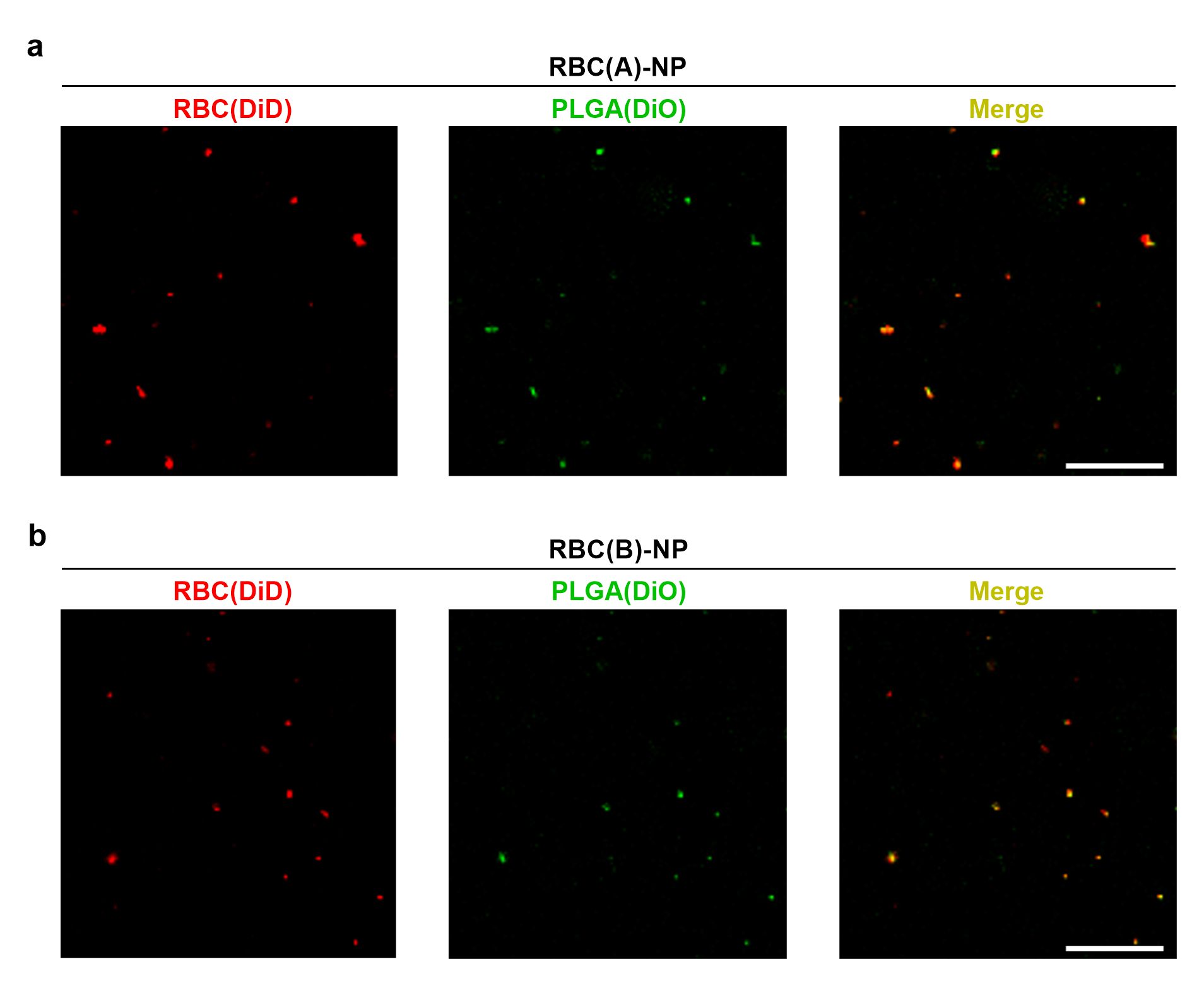


**Figure S3.** Confocal fluorescence microscopy images of dual-dye-labeled (a) RBC(A)-NP and (b) RBC(B)-NP (DiD labeled-RBC membrane, red color; DiO labeled-PLGA core, green color). Scale bar = 20 μm.

**Figure S4. Antigen A and B on the respective RBC(A)-NP and RBC(B)-NP determined by Western blot**

The A and B antigens on the respective RBC(A)-NP and RBC(B)-NP was characterized by Western blot. Briefly, 20 μL of samples containing RBC(A) membrane, RBC(A)-NP, and RBC(B)-NP at an equivalent concentration of 1 mg/mL were mixed with 5× loading buffer, followed by heating at 90 °C for 10 min. The heated samples were added to a 15-well Blot 10% SDS-PAGE gel and run at 150 V for 50 min. Following the electrophoresis, the protein were translocated onto the PVDF membranes at 150 mA for 45 min, then the PVDF membranes were blocked with 5% skimmed milk for 120 min, followed by washing with 1× TBST thricely. After that, the PVDF membranes were incubated with anti-A (1: 2000 dilution) or anti-B (1: 2000 dilution) primary antibodies overnight at 4 ℃, followed by washing unbound antibodies away using 1× TBST for three times. Then, the primary antibody-coated PVDF membranes were incubated with HRP-conjugated IgG secondary antibody (1: 10000 dilution) for another 1 h at RT. The resultant membranes were washed with 1 × TBST three times. The blot signals in membranes were visualized by an ECL detection kit and were photographed by a ChemiDoc CRS imaging system (Bio-Rad, USA).


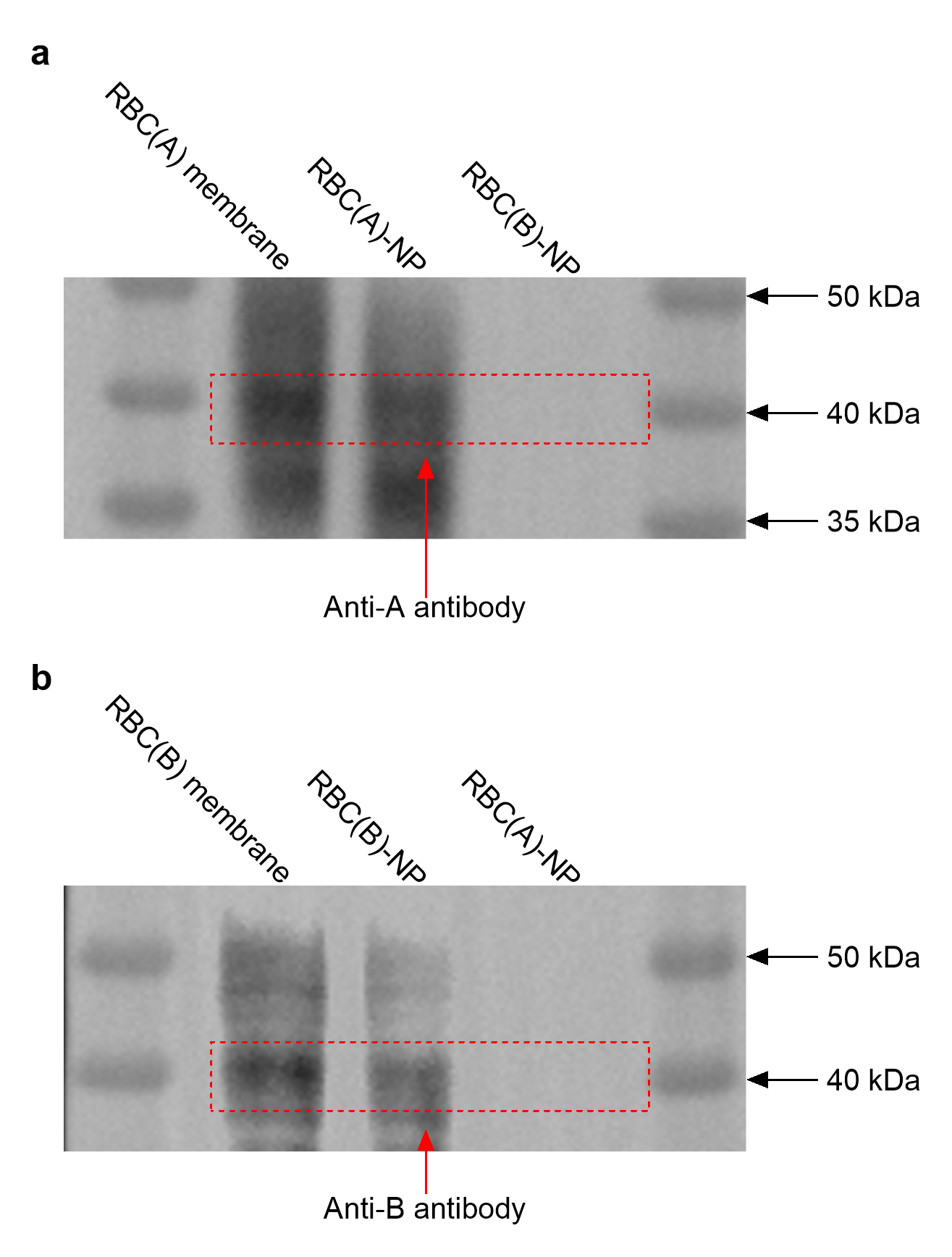


**Figure S4.** Antigen A (38 kDa) expressed in RBC(A) membrane and RBC(A)-NP or antigen B (40 kDa) expressed in RBC(B)-NP as determined by Western blot.

**Figure S5. Morphology of RBC-NP following the 7 days store in 10% FBS**


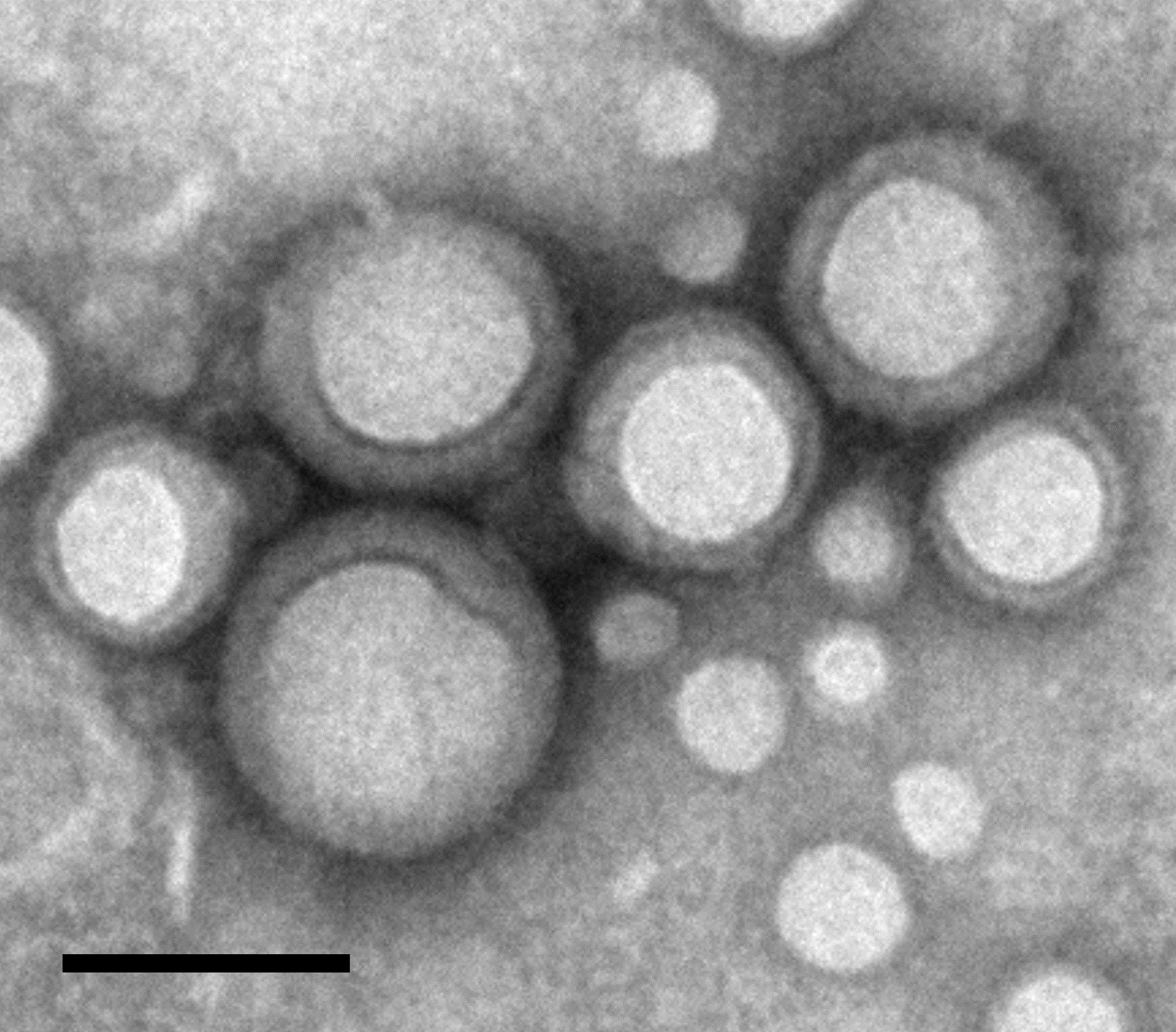


**Figure S5.** Morphology of RBC-NP after being dispersed in 10% FBS for 7 days, as visualized by TEM. Bar = 200 nm.

**Figure S6. RBCs agglutination scoring determined by CTT**

The conventional tube technique (CTT) was used to determine the blood agglutination scoring in the study. At first, packed RBC(A)s or RBC(B)s and their corresponding IgM serums (pre-determined at 1: 256 titers) were collected from A or B whole blood according to the section ‘*Derivation of human RBC membrane*’ in Method part of the main text. After that, 50 μL of 5 vol% RBC(A)s or RBC(B)s (0.55 vol% to a final volume of 450 μL) were placed into tubes and followed by the addition of varying dilutions of anti-A or anti-B IgM serums to a final volume of 450 μL. After incubation for 20 min at RT, the samples were subjected to centrifugation of 2,000 rpm for 1 min. Finally, the RBC samples in tubes were slightly shaken and photographed under white light. Only RBC(A)s or RBC(B)s were used as the negative controls. The degrees of RBC clumps were scored regarding clump volume/number as below: 0, without observable clump; 0.5, rare dispersed clumps; 1, small dispersed clumps; 1.5, small condensed clumps; 2, moderate condensed clumps; 2.5, moderately condensed clumps with small dispersed clumps; 3, big clumps with many spread small clumps; 3.5, big clumps with few scattered small clumps; 4, big condensed clumps.


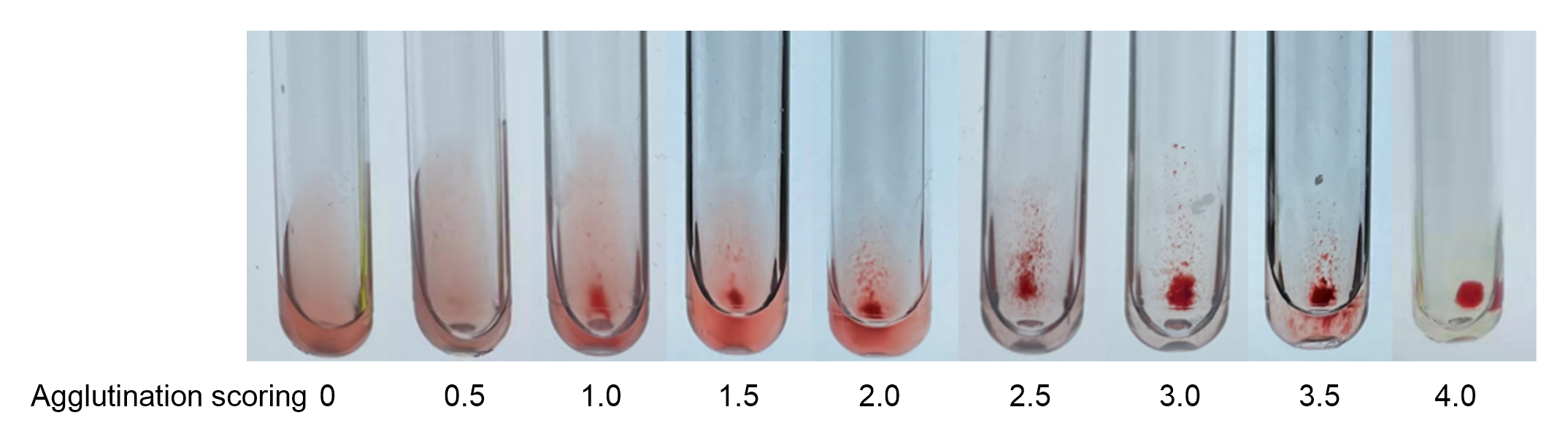


**Figure S6.** Images showing the different degrees of RBCs agglutinations for scoring evaluation.

**Figure S7. The neutralization correlation between RBC-NPs and their corresponding IgM antibodies**

To correlate the neutralization relevance between RBC-NPs and their corresponding IgM antibodies, various concentrations of RBC(A)-NP (0, 0.36, 0.7, 1.4, 2.8, and 5.6 mg/mL) or RBC(B)-NP (0, 0.36, 1.4, 2.8, and 5.6 mg/mL) were respectively mixed with 11 vol% of anti-A (1: 128 titers) or anti-B IgM serums (1: 128 titers) and incubated for 20 min at RT, followed by centrifugation at 13,000 g for 10 min. Afterward, the supernatants were collected and subjected to IgM ELISA measurements.


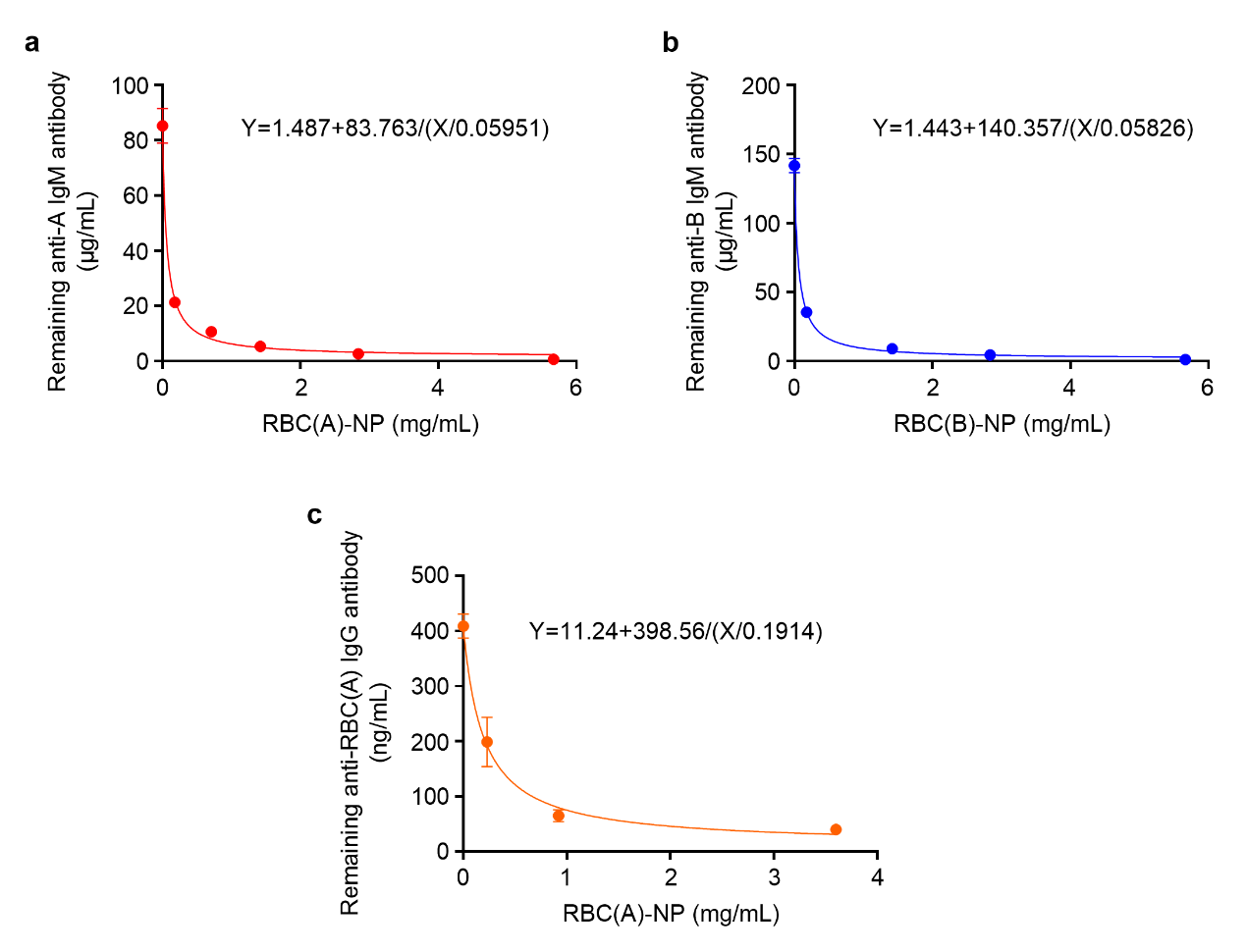


**Figure S7.** Neutralization curves of RBC(A)-NP (a) or RBC(B)-NP (b) dosages against the corresponding fixed volumes of anti-A IgM or anti-B IgM. n = 3, mean ± sd.

**Figure S8. Combination of RBC(A)-NP and RBC(B)-NP to overcome incompatible blood transfusion between wRBC(O) and RBC(AB)s**

To simultaneously neutralize anti-A&B IgM antibodies in wRBC(O) for RBC(AB)s transfusion, the experimental wRBC(O) (4.4 vol% of RBC(O)s to a final volume of 450 μL) was pre-mixed with RBC(A)-NP and RBC(B)-NP at a respective concentration of 2.8 mg/mL and incubated for 20 min at RT. Following incubation, the samples were subjected to the addition of 50 μL 5 vol% RBC(AB)s to a final volume of 450 μL and incubated for another 20 min at RT. Afterward, the mixtures were centrifuged at 2,000 rpm for 1 min, and the agglutination scorings were determined after slight shaking. The experimental wRBC(O) treated with 0.55 vol% of RBC(AB)s alone or pre-treated with either 2.8 mg/mL of RBC(A)-NP or 2.8 mg/mL of RBC(B)-NP and followed by adding 0.55 vol% of RBC(AB)s were used as controls.


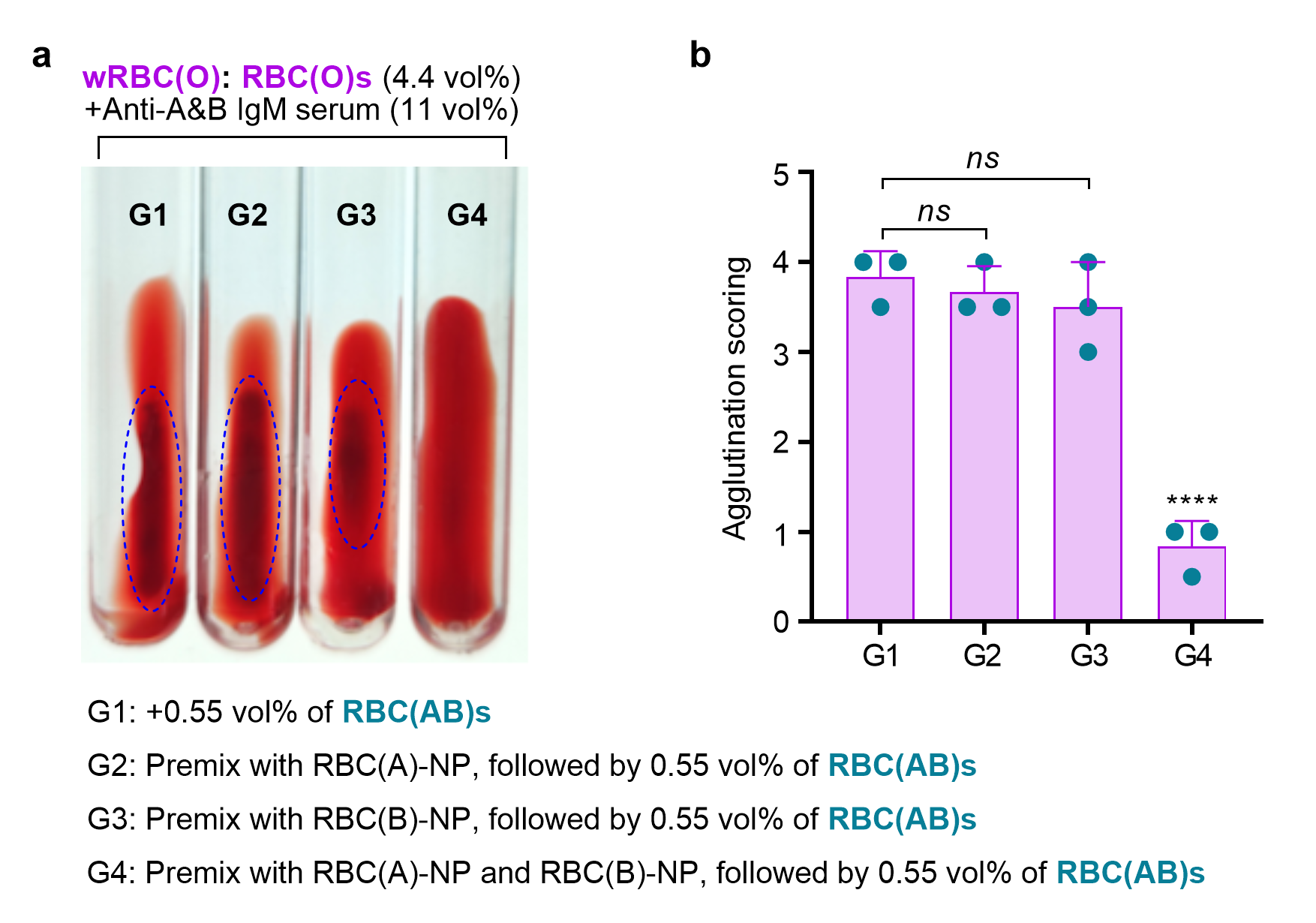


**Figure S8.** (a) Images showing an experimental wRBC(O) pre-incubated with RBC(A)-NP and RBC(B)-NP at respective concentrations of 2.8 mg/mL was successfully transfused with 0.55 vol% of RBC(AB)s. The experimental wRBC(O) treated with 0.55 vol% of RBC(AB)s alone or pre-treated with 2.8 mg/mL of RBC(A)-NP or RBC(B)-NP and followed by adding 0.55 vol% of RBC(AB)s were used as controls. (b) Statistical analysis of agglutination scoring for (a). (n = 3, mean ± sd).

**Figure S9. The neutralization correlation between RBC-NP and its corresponding IgG antibody**

To correlate the neutralization relevance between RBC-NP and their corresponding IgG antibodies, various concentrations of RBC(A)-NP (0, 0.23, 0.92, and 3.6 mg/mL) were mixed with 50 μL of pooled anti-RBC(A) IgG serums (10 vol%) to a final volume of 500 μL. After 2 h incubation at RT, the samples were centrifuged at 13,000 g for 10 min, followed by harvesting the supernatants for IgG quantification using an IgG ELISA kit.


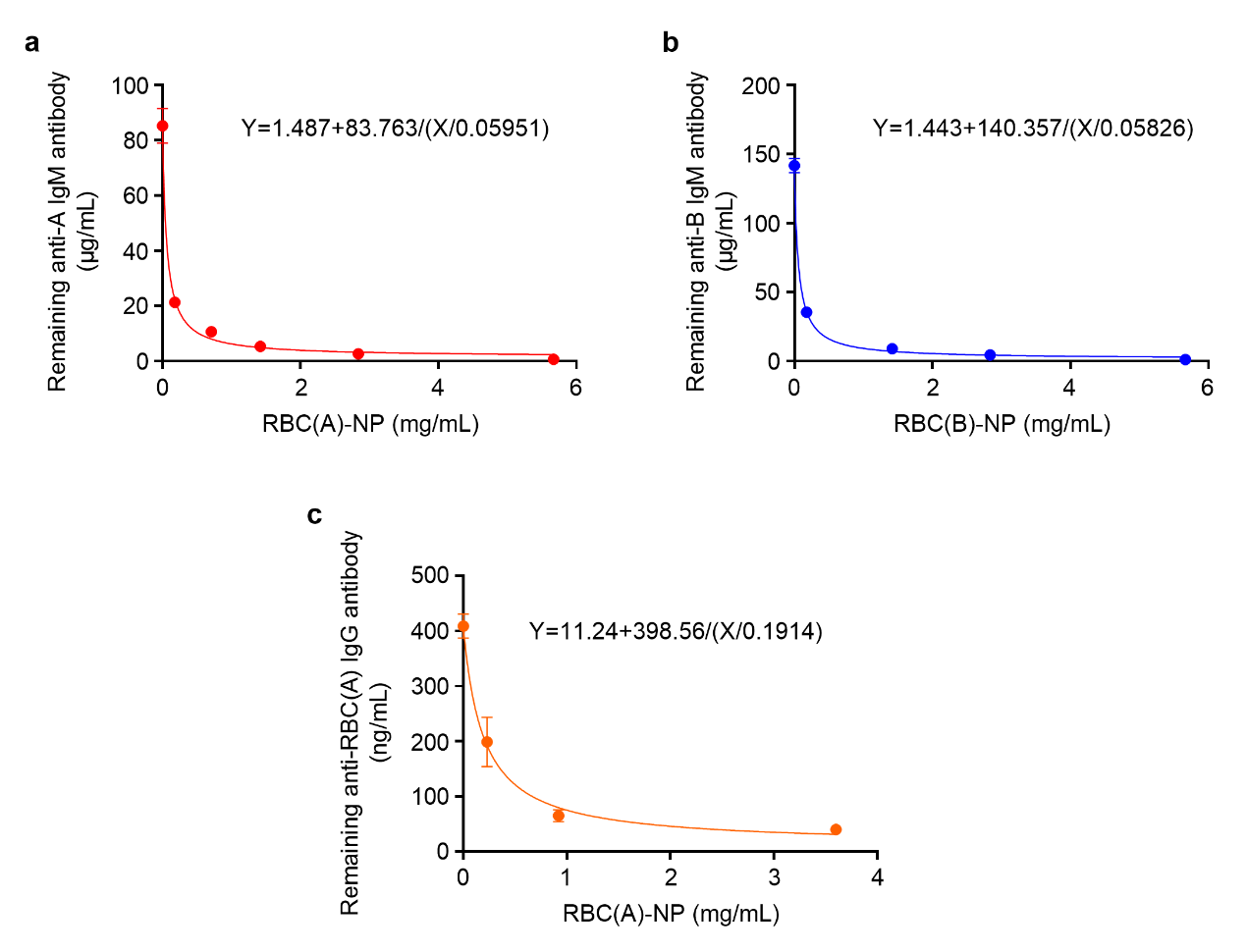


**Figure S9.** Neutralization curves of RBC(A)-NP dosages against the corresponding fixed anti-RBC(A) IgG volume. n = 3, mean ± sd.

**Figure S10. Determination of antigen A in eRBC(A)-NP before and after the lyophilization treatment**

Protein compositions of the fRBC(A)-NP, eRBC(A)-NP, and eRBC(A)-NP after lyophilization were characterized by SDS-PAGE. Briefly, 20 μL of each sample at an equivalent protein concentration of 1 mg/mL were mixed with 5× loading buffer, followed by heating at 90 °C for 10 min. The heated samples were added to a 15-well Blot 10% SDS-PAGE gel and run at 150 V for 50 min. Following electrophoresis, the gel was stained with Coomassie Blue and photographed. Meanwhile, the samples loaded SDS-PAGE gel was subjected to the Western blot treatment according to the method in the part of ‘**Supplementary Fig. 4**.’


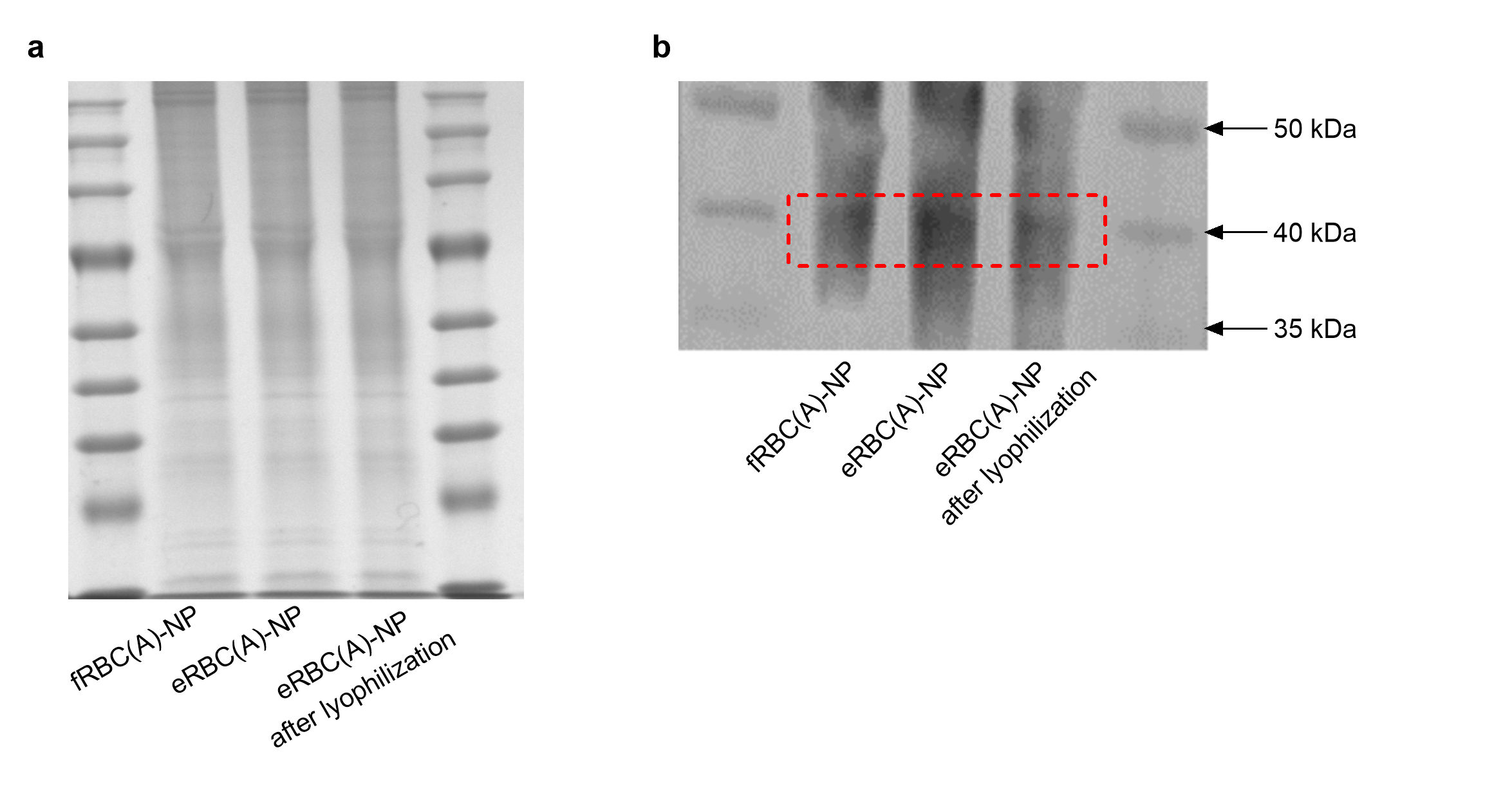


**Figure S10.** SDS-PAGE showing protein compositions (a) and Western blot presenting specific antigen A (b) of fRBC(A)-NP, eRBC(A)-NP, and eRBC(A)-NP after treatment of lyophilization and resuspension.

**Figure S11. Determination of circulation property of eRBC(A)-NP in mice**

DiD dye-labeled eRBC(A)-(DiD)NP and fRBC(A)-(DiD)NP at 50 mg/kg were administrated to mice. After particles were given, the 30 μL of blood sample was harvested at planned time points (1, 3, and 30 min, and 1, 3, 7, 24, 48, and 72 h) via submandibular puncture. Then, blood at 30 μL was added to 70 μL water for fluorescence measurement at an excitation/emission of 630/670 nm using a plate reader.


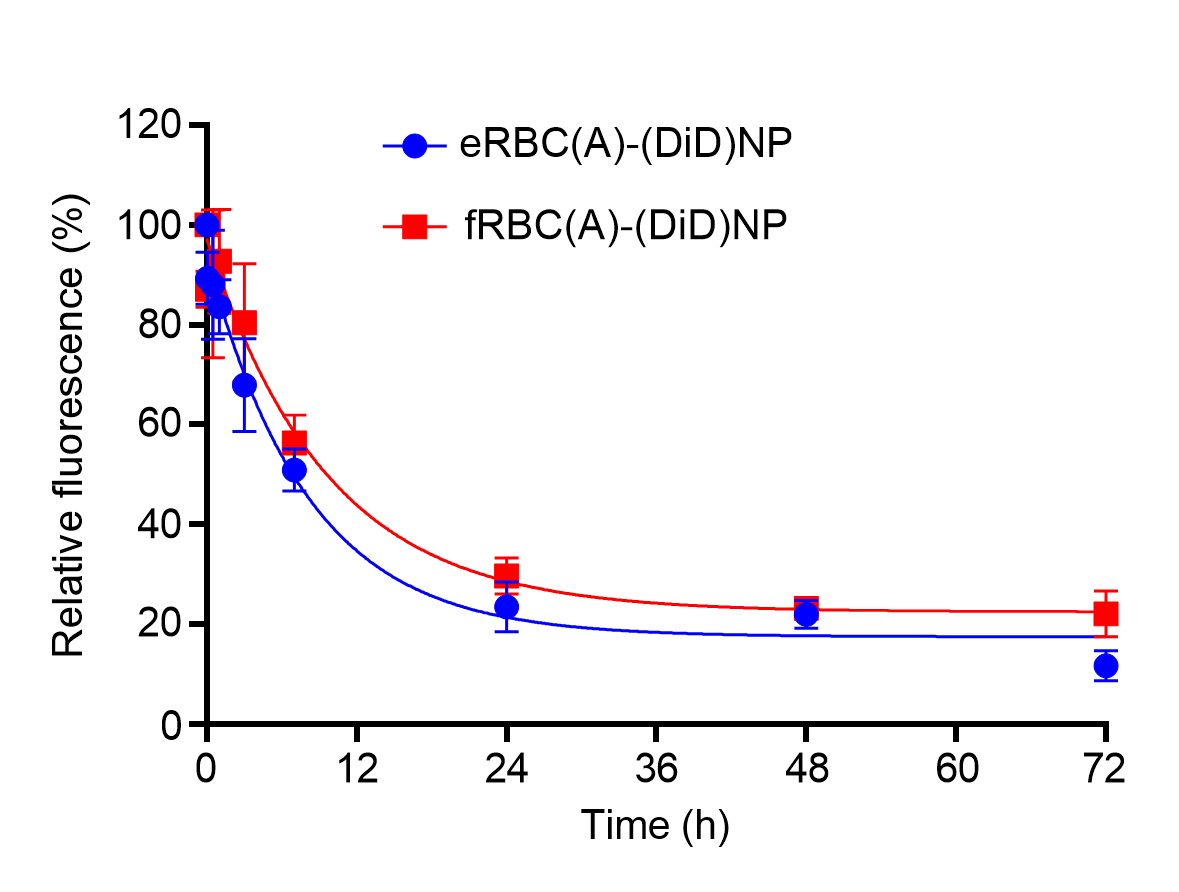


**Figure S11.** Relative fluorescence signal changes of eRBC(A)-(DiD)NP and fRBC(A)-(DiD)NP in circulation over time after intravenously administering at a fixed dose of 50 mg/kg into mice. n = 5, mean ± sd.

**Figure S12. Biodistribution of the antibodies-captured RBC-NP at the first 0.25, 1, and 3 h after given in normal mice**


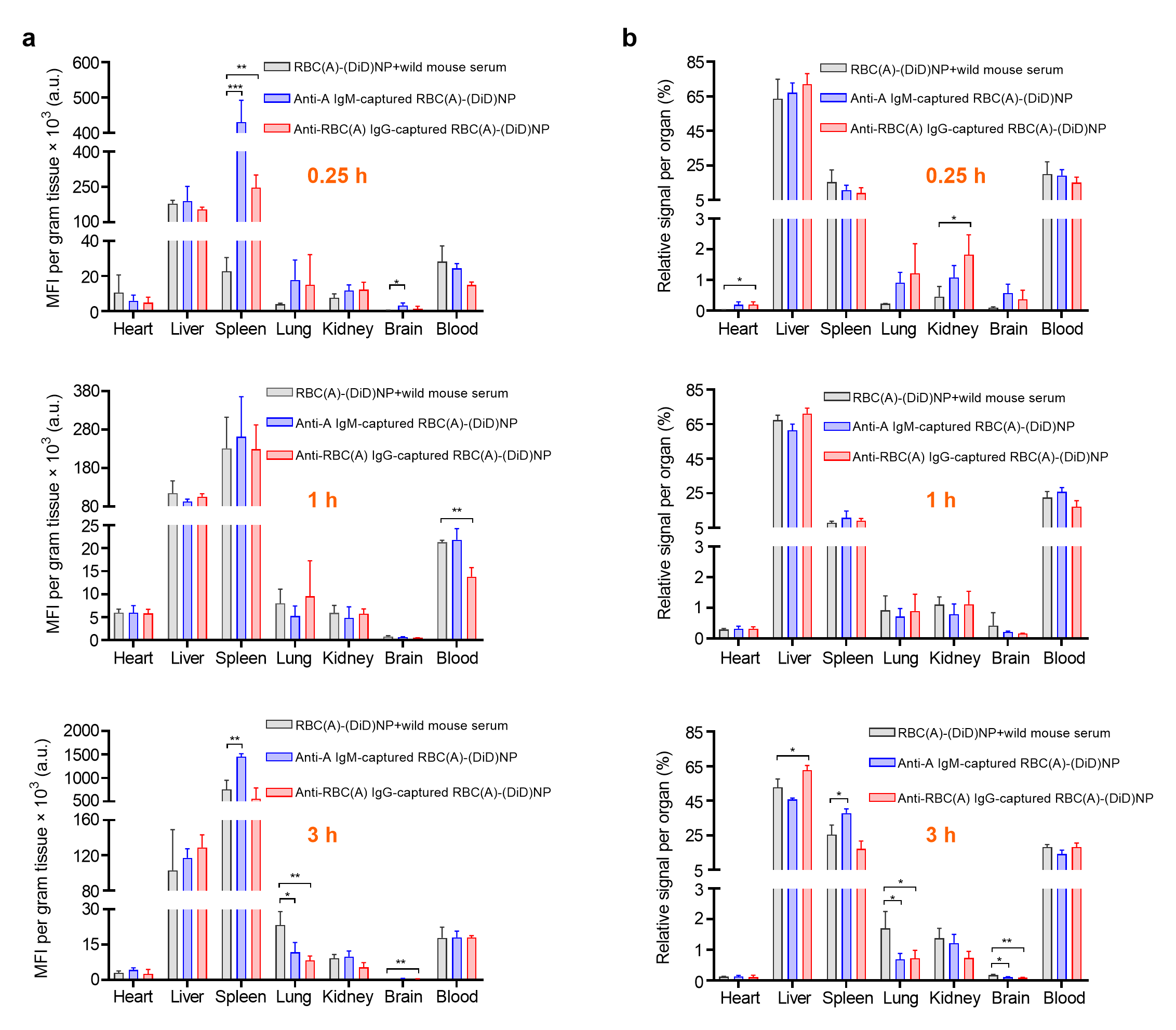


**Figure S12.** Fluorescence intensity per gram of tissue (a) or relative signal per organ (b) for dissected major organs from mice 0.25, 1, and 3 h after intravenous given of anti-A IgM or anti-RBC(A) IgG-captured RBC(A)-(DiD)NP in the hemorrhagic shock mice. RBC(A)-NP pretreated with wild mouse serum was used as a control. Mice treated with PBS only were served as the negative control. n = 3, mean ± sd.

**Figure S13. IgM or IgG-sequestered RBC-NP uptake by macrophages *in vitro***

RAW264.7 cells at a density of 4× 10^4^ per well were incubated in 24-well plates (DMEM supplemented with 10% FBS) for 24 h. Meanwhile, DiD-labeled RBC(A)-(DiD)NP at 2.8 mg/mL was incubated with 11 vol% of anti-A IgM serum (1: 128) or at 3.6 mg/mL was incubated with 10 vol% of anti-RBC(A) IgG serum (pooled) for 1 h at RT. The resultant mixtures were subjected to centrifugation at 13,000 g for 10 min. After centrifugation, the pelleted nanoparticles were redispersed in PBS and transferred to RAW264.7 cells. At set time points incubation (1, 2, and 3 h), the RAW264.7 cells were washed with PBS three times and then digested to single cell suspensions for flow cytometry measurements using the Cytoflex (Beckman. USA). In parallel, fluorescent images of RAW264.7 cells at the same time points were photographed using fluorescence microscopy. RBC(A)-(DiD)NP pretreated with wild mouse serum and then incubated with cells was used as a control.


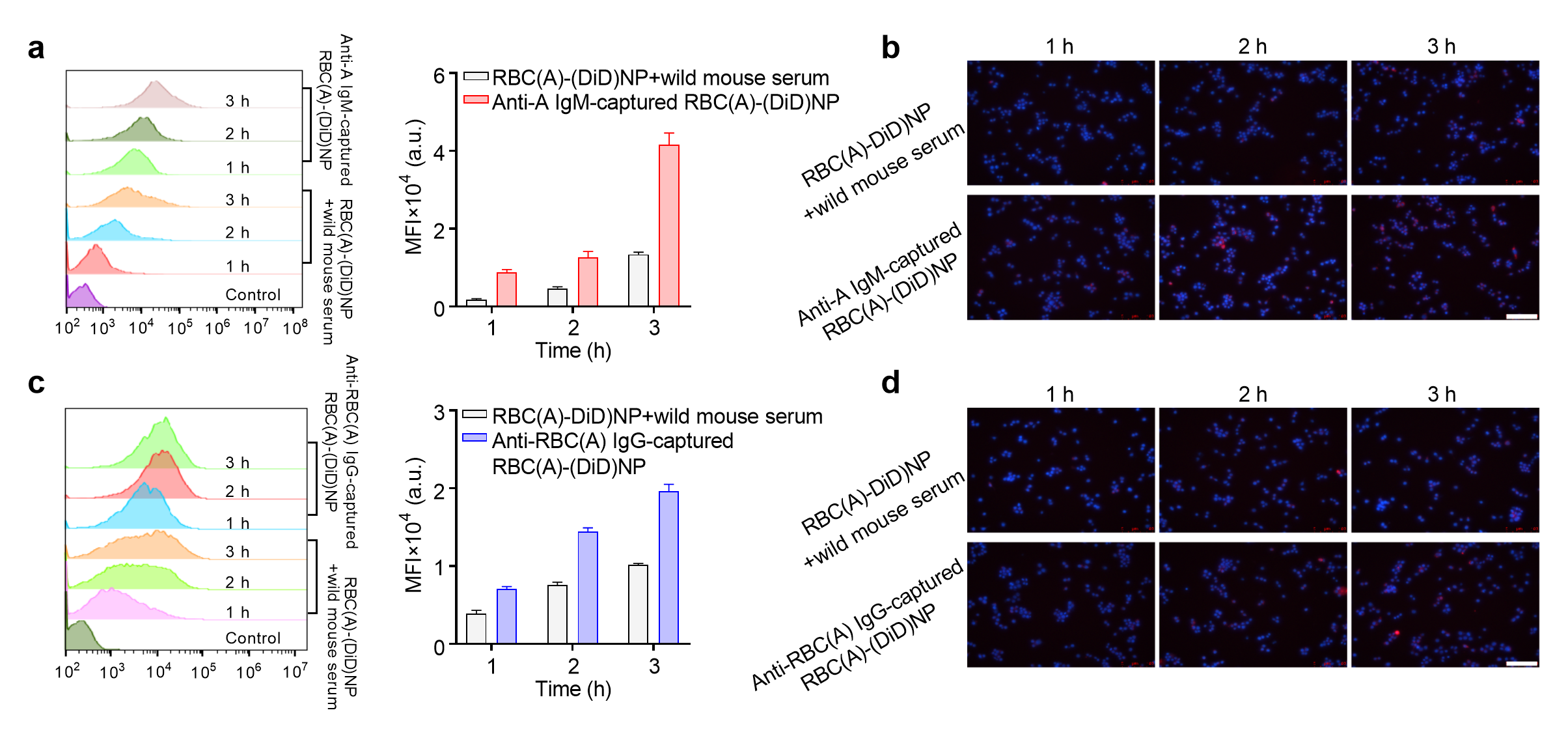


**Figure S13.** (a) Flow cytometry analysis for internalization ability of anti-A IgM-sequestered RBC(A)-(DiD)NP in RAW264.7 cells at different time points (1, 2, and 3 h). RBC(A)-(DiD)NP pretreated with wild mouse serum and then incubated with cells was used as a control. (n = 3, mean ± sd). (b) The corresponding representative fluorescent images for (a) at the same time points. Scale bar = 100 μm. (c) Flow cytometry analysis for internalization ability of anti-RBC(A) IgG-sequestered RBC(A)-(DiD)NP in RAW264.7 cells at different time points (1, 2, and 3 h). RBC(A)-(DiD)NP pretreated with wild mouse serum and then incubated with cells was used as a control. (n = 3, mean ± sd). (d) The corresponding representative fluorescent images for (c) at the same time points. Scale bar = 100 μm.

**Figure S14. Biocompatible assessment of antibody-captured RBC-NP at a higher dose of 200 mg/kg in hemorrhage shock murine model**

The biocompatibility of anti-A IgM or anti-RBC(A) IgG-captured RBC(A)-(DiD)NP at a higher dose of 200 mg/kg in hemorrhagic shock mice was evaluated. Briefly, a volume-targeted hemorrhagic shock murine model was first constructed by taking 300 μL of blood from the C57/B6 mouse via submandibular puncture. At 15 min following the blood loss, the mice were intravenous injected with anti-A IgM or anti-RBC(A) IgG-captured RBC(A)-NP (200 mg/kg). At 24 h after treatments, the whole blood samples were collected through the submandibular puncture and then subjected to detect blood routine parameters by Hematology Analyzer (Biote, HL-2400). Meanwhile, the blood samples were centrifuged at 1,000 g for 5 min, and the collected serums were tested by Beckman Coulter (AU480) for biochemistry parameters analysis.


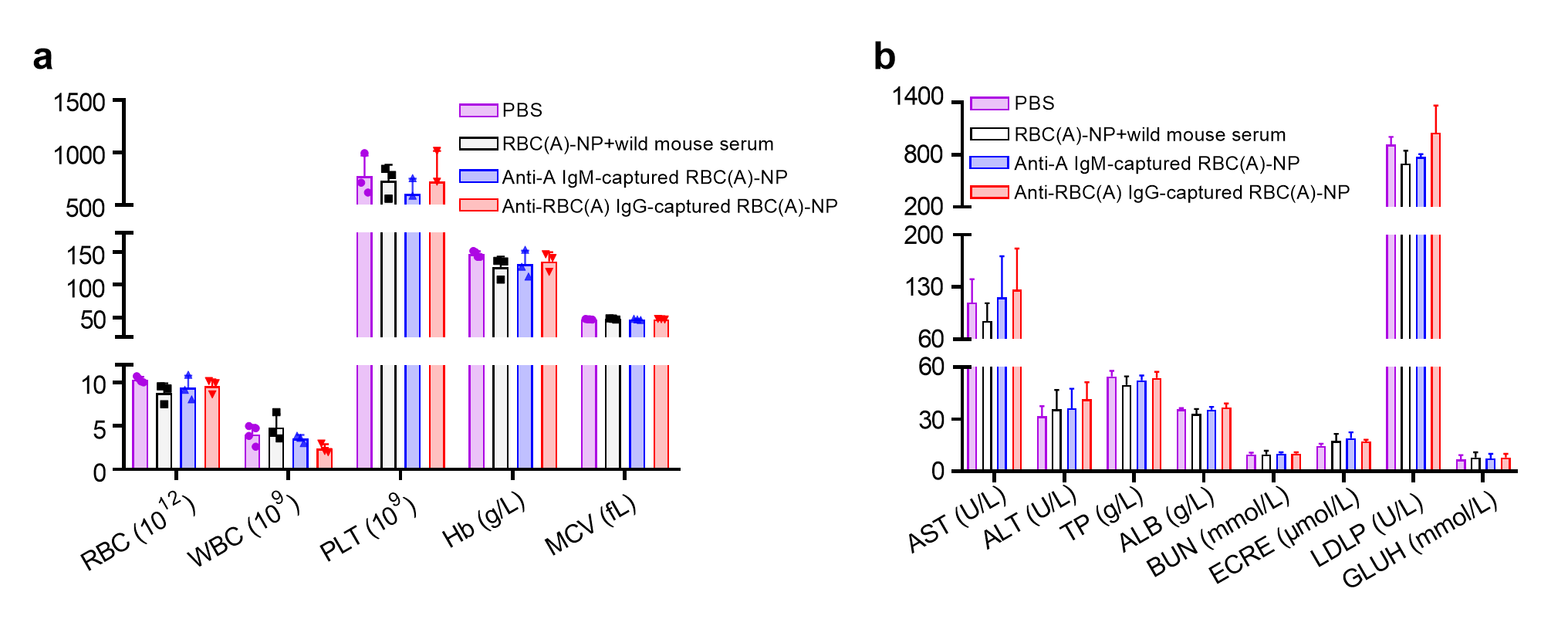


**Figure S14.** (a) Counts of various blood cells 24 h after intravenous injection of four different treatments. (n = 3, mean ± sd). RBC, red blood cell; WBC, white blood cell; PLT, platelet; Hb, Hemoglobin; and MCV, mean corpuscular volume. (b) Blood chemistry panel has taken after intravenous injection of four different treatments. (n = 3, mean ± sd.). AST, aspartate aminotransferase; ALT, alanine aminotransferase; TP, total protein; ALB, albumin; BUN, blood urea nitrogen; CRE, creatinine; LDLP, low density lipoprotein; and GLUH, glutamic acid.

**Figure S15. Kinetic efficacy of RBC-NP for IgG neutralization *in vivo***

The C57/B6 mice were firstly pretreated with 100 μg of poly(I:C) per mouse by intraperitoneal, followed by intravenously injecting 12.5 μL of human RBC(A)s afterward. At 2 weeks after treatment, mice were intravenous injected with RBC(A)-NP at 100 mg/kg, followed by collecting whole blood at various time points (1 min, and 6, 12, 24, 48, 72, 96, and 120 h) for anti-RBC(A) IgG amounts quantification. After removing blood cells, the anti-RBC(A) IgG in the remaining serum was tested using ELISA assay. Briefly, 96-well plates were pre-coated with RBC(A) membrane (20 μg/mL, 100 μL per well) using 5× ELISA coating buffer at 4 °C overnight. After removal of free membrane, the wells were blocked with 1% BSA for 1 h at RT. Then, 100 μL of serums (1: 100 dilution) were added and incubated for 1 h at RT. After washing with PBS, the HRP-conjugated secondary antibody was supplemented and incubated for another 1 h at RT. After that, the wells were washed with PBS three times and developed with 100 μL per well of TMB substrate for 15 min. Then, the reaction was terminated by adding 100 μL of stop solution. Finally, the absorbance at 450 nm was measured with a plate reader (SpectraMax I3 MD USA). Serums collected from mice received 100 μg poly(I:C) and 50 μL of human RBC(A)s were used as the positive control (PC).


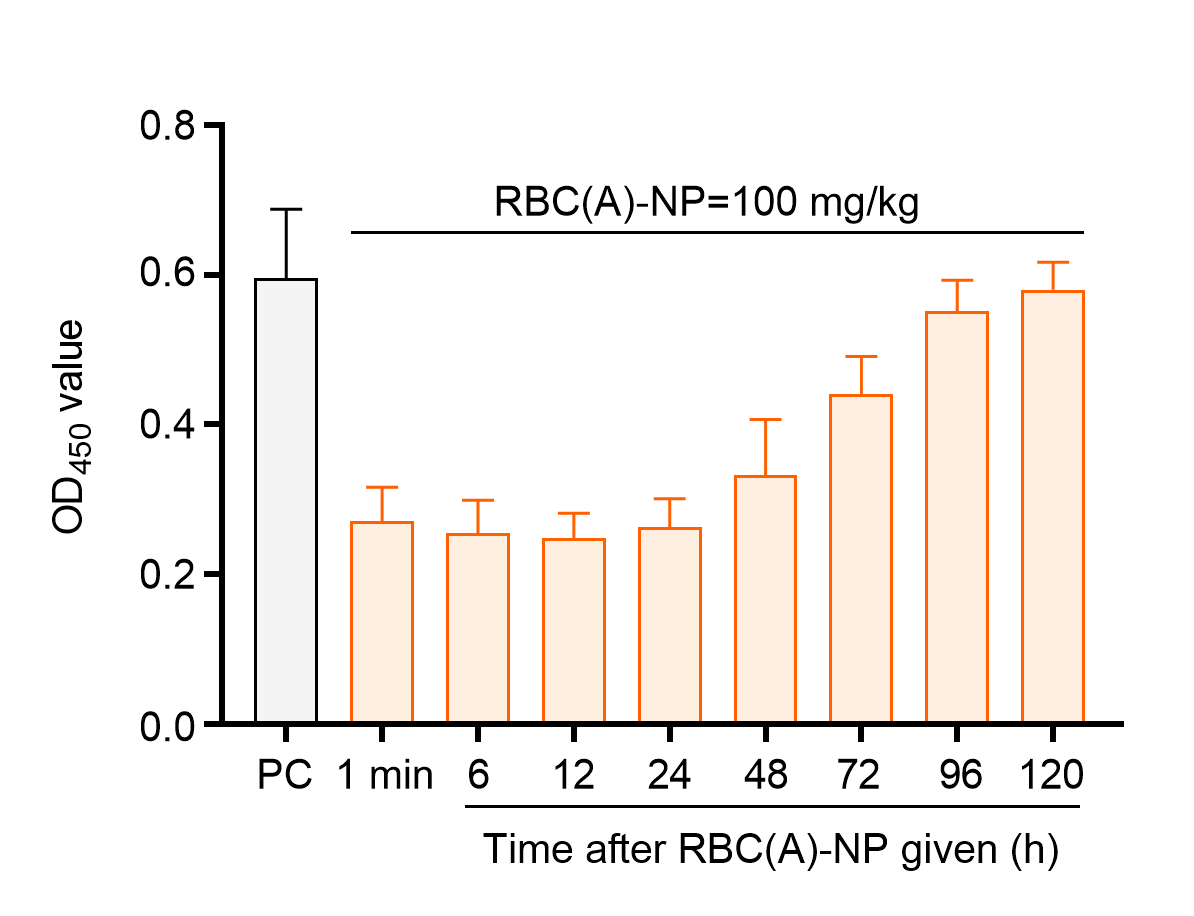


**Figure S15.** Remaining anti-RBC(A) IgG in mouse serum as expressed by OD_450_ values at different time points (1 min, and 6, 12, 24, 48, 72, 96, and 120 h) after treatment with RBC(A)-NP. RBC(A)s immunized mice without RBC(A)-NP treatment was used as the positive control (PC). n = 3, mean ± sd.

**Supporting video. RBC(A)-NP blocks the anti-A IgM antibody-mediated RBC(B)s agglutination**
